# Phytoplasma-Associated Diseases in South America: Thirty Years of Research

**DOI:** 10.3390/microorganisms12071311

**Published:** 2024-06-27

**Authors:** Helena Guglielmi Montano, Assunta Bertaccini, Nicola Fiore

**Affiliations:** 1 Department of Entomology and Plant Pathology, Federal Rural University of Rio de Janeiro (UFRRJ), Seropédica 23897-000, Brazil; hgmontano@yahoo.com.br; 2 Department of Agriculture and Food Science, Alma Mater Studiorum—University of Bologna, 40127 Bologna, Italy; 3 Department of Plant Health, Faculty of Agricultural Sciences, University of Chile, Santiago 8820808, Chile; nfiore@uchile.cl

**Keywords:** plant disease, insect vector, epidemiology

## Abstract

Phytoplasma-associated diseases are mainly insect-transmitted and are present worldwide. Considering that disease detection is a relevant environmental factor that may elucidate the presence of these diseases, a review reporting the geographic distribution of phytoplasma taxa in geographically consistent areas helps manage diseases appropriately and reduce their spreading. This work summarizes the data available about the identification of the phytoplasma associated with several diverse diseases in South America in the last decades. The insect vectors and putative vectors together with the plant host range of these phytoplasmas are also summarized. Overall, 16 ‘*Candidatus* Phytoplasma’ species were detected, and those most frequently detected in agricultural-relevant crops such as corn, alfalfa, grapevine, and other horticultural species are ‘*Ca*. P. pruni’, ‘*Ca*. P. asteris’, and ‘*Ca.* P. fraxini’.

## 1. Introduction

During the last decades, the information about the species belonging to the genus ‘*Candidatus* Phytoplasma’ infecting different crops in South America has increased, offering important indications about the phytoplasma-associated diseases present in the region. Some of the detected phytoplasmas are considered native to South America, having been identified exclusively or prevalently in this geographical area.

Phytoplasmas are bacteria that inhabit plant sieve tubes and are associated with diseases in more than one thousand plant species [1]. They are mainly transmitted by phloem-feeding insect vectors belonging to the Cicadellidae (leafhoppers), Cixiidae (planthoppers), and Psyllidae (psyllids) families. Phytoplasma identification is based on 16S rRNA gene sequence, in some cases complemented by using selected housekeeping genes. The classification of these pathogens is constantly in progress, and since it is not easy to cultivate them in vitro, the mostly used identification tools are the molecular ones. The phytoplasma identification is reported as ‘*Candidatus* Phytoplasma’, in which more than 50 taxa are officially described, mainly based on identity percentages in the 16S rRNA gene and/or ANI values (below 98.65% and 95% respectively) [2,3], and/or as ribosomal group and subgroup [4] and amended from published data when necessary and possible.

The present work focuses on the main diseases associated with phytoplasmas in different crops of importance for South America, providing updated information about the presence of diverse symptomatology associated with typical symptoms [5]. From this overview, it appears that the phytoplasmas associated with diseases are in a strong relationship with the diverse environments, the insect transmission, the biological characteristics and/or the human agricultural activities. Therefore, the information about the identity and localization of the diverse phytoplasmas in South American countries allows their focused management and containment of their spreading in economically and ecologically relevant plant species. The known and putative insect vectors are also reported together with alternative plant host species of the phytoplasmas associated with the most important diseases detected in the diverse countries of South America.

## 2. ‘*Candidatus* Phytoplasma asteris’, ‘*Ca*. P. tritici’, and ‘*Ca*. P. lycopersici’

Cereals, fruit crops, industrial crops, ornamental and vegetable species, and weeds have been reported as hosts for phytoplasmas in ribosomal subgroups 16SrI-A and -B (‘*Ca*. P. asteris’), 16SrI-C (‘*Ca*. P. tritici’), and 16SrI-Y (‘*Ca*. P. lycopersici’), in several South America countries [3].

### 2.1. Bolivia

The diseases named “hoja de perejil” of tomato (*Solanum lycopersicum* L.) and “brotes grandes” of potato (*Solanum tuberosum* L.) were detected in Bolivia about 23 years ago. Alfalfa (*Medicago sativa* L.) witches’ broom and little leaf of the native weeds *Morrenia variegata* (Griseb.) T. Mey and mora-mora (*Serjania perulacea* Radlk) were also observed in the vicinity of the production fields. Aster yellows group (16SrI) phytoplasmas were identified in potato, alfalfa, and mora-mora plants, which became infected with 16SrI-B strains; meanwhile, “hoja de perejil” and morrenia little leaf-associated phytoplasma strains shared 97.5% of 16S rRNA gene sequence identity with ‘*Ca*. P. asteris’, and for this reason, they were enclosed in the separate ‘*Ca.* P. lycopersici’ taxon (16SrI-Y) [6,7].

### 2.2. Brazil

Corn production in Brazil has grown rapidly over the last decades, and one of the big drivers has been the introduction of “safrinha”, or second crop, corn (https://www.vantrumpreport.com/2020/06/23/a-brief-history-of-brazils-little-corn-crop/ (accessed on 12 February 2024). With the main crop followed by the “safrinha”, corn plants are present in the field for almost the whole year, and this has enhanced the occurrence and the incidence of maize bushy stunt (MBS) disease, which is among the first phytoplasma diseases recorded in Brazil [8]. The molecular identification of a 16SrI phytoplasma associated with the disease was demonstrated more than two decades after the initial reports, [9] and nowadays, this is one of the most important phytoplasma diseases in the country and it is among the major corn diseases. The well-known insect vector for the MBS phytoplasma, the leafhopper *Dalbulus maidis* (DeLong and Wolcott) (Hemiptera, Cicadellidae), is transmitting the phytoplasma in Brazil and is widespread throughout the maize-producing areas [10]. In “safrinha” cultivations, the incidence of the corn stunt complex (MBS phytoplasma and corn stunt spiroplasma) increases and there is a reduction in grain yield [11]. According to Oliveira and Frizzas [12], the changes in the corn production system experienced in recent years, with increasing planted areas, wide sowing windows, diversification of sowing seasons, and dissemination of volunteer maize plants, are a consolidated reality. Unfortunately, these conditions greatly favor the maintenance and, in many cases, the increase in the population of the insect vector by reducing the maize off-season periods. Off-season maize is the main modulator of *D. maidis* populations as this species depends on maize for reproduction.

Sugarcane is affected by the sugarcane yellow leaf (SCYL) syndrome, which is recorded in several countries, and in Brazil, it is particularly important in the state of São Paulo. The 16SrI-B phytoplasma was found in association with SCYL. Although it is endemic to the state of São Paulo, it is considered a potentially important disease because it can lead to the substitution of susceptible sugarcane varieties [13].

Oil palm (*Elaeis guineensis* Jacq.) was introduced in Brazil from Africa and is well adapted to the northeast region of the country. According to the Brazilian Palm Oil Producer Association (Abrapalma), over 85% of the country’s production is concentrated in the state of Pará. Oil palm has been affected by fatal yellowing disease (FY), which is responsible for decimating thousands of plants in the state of Pará [14] and severely affecting oil palm plantations in Latin America [15]. The etiology of FY in Brazil has been searched since the first reports of the disease in the country, and so far, in case it is of biotic origin, no causal agent has been attributed to it. Despite the fact that phytoplasmas affiliated with group 16SrI have been found associated with oil palm samples from diseased plants, further corroborated by other studies [16,17], the hypothesis proposing phytoplasma as associated with FY was discarded because very few samples tested positive in the PCR assays and the attempt to reproduce the disease by grafting did not yield diseased plants [15].

Soybean is a main crop in Brazil, and it shows delayed senescence due to abiotic and biotic factors, including feeding from insects and/or infection by viruses and phytoplasmas, environmental conditions, and host genetics. In 2017, Pereira and Bedendo [18] reported the occurrence of a soybean delayed maturity disease associated with phytoplasmas in the state of Mato Grosso, the main soybean-producing state in Brazil. Diseased plants exhibit pods of reduced size, reduced number of grains per pod, green stems, and retained leaves by the end of the cropping cycle. The phytoplasma detected in diseased plants is affiliated with subgroup 16SrI-B, for which a 16S rRNA gene sequence (strain Soy-BrI) was deposited in GenBank under the accession number (acc. no.) JQ065052.

Molecular analysis based on conventional and virtual RFLP patterns and similarity coefficient calculations identified the phytoplasma strain BSP-Br1 [GenBank acc. no. EU423898], associated with *Bougainvillea spectabilis* shoot proliferation as belonging to subgroup 16SrI-B. The phytoplasma was detected in bougainvillea-potted plants displaying foliar chlorosis, shoot proliferation, leaf and bract deformations, and decline in commercial nurseries in the state of São Paulo [19]. A phytoplasma affiliated with subgroup 16SrI-B was identified in broccoli stunt disease from São Paulo state and designated broccoli stunt phytoplasma BSP-3 (GenBank acc. no. JX626330) [20,21]. Also, stunt disease of cabbage was reported in association with a phytoplasma belonging to group 16SrI, but no subgroup has been assigned to the pathogen [22]. Diseases associated with phytoplasmas affiliated with group 16SrI have been reported in plant species belonging to distinct botanical families. However, some of them are not completely described as the phytoplasma reported in plants of *Erigeron bonariensis* L. with symptoms of witches’ broom [23], the one associated with grapevine yellows [24] and with strawberry phyllody [25].

Sequencing analysis of phytoplasmas from distinct plant hosts indicates the presence, in addition to 16SrI-B, of other phytoplasma subgroups within group 16SrI. *Waltheria indica* L. witches’ broom phytoplasma (WIWB) (GenBank acc. no. KX691443) [26] and the phytoplasma detected in *Macroptilium lathyroides* L. yellow leaf (MLYL) disease (GenBank acc. no. KY270560) [27] were identified as members of the 16SrI-S subgroup. These phytoplasma strains are now classified as ‘*Ca.* P. tritici’ and were obtained from the respective plants from the municipality of Vila Velha, in the Espírito Santo state. A phytoplasma affiliated with subgroup 16SrI-S (GenBank acc. no. KF878383) was also identified in sun hemp (*Crotalaria juncea* L.) plants with witches’ broom in the state of São Paulo [28].

### 2.3. Chile

**Figure 1:**
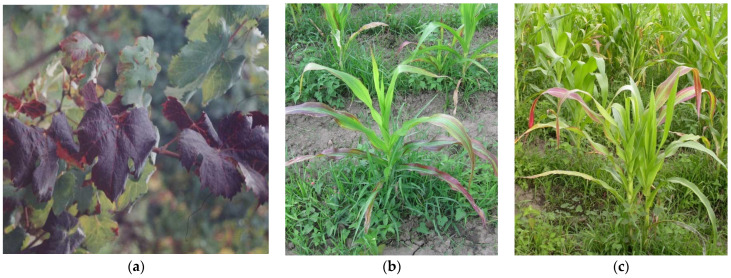
Plants infected with ‘*Ca*. P. asteris’ (16SrI-B): in (**a**) grapevine leaves showing reddening and downward curling in Chile; in (**b**,**c**), corn plants showing reddening of young leaves in Colombian cultivations.

Grapevine yellows disease associated with ‘*Ca.* P. asteris’ (16SrI-B) and ‘*Ca*. P. tritici’ (16SrI-C) was detected in Chilean vineyards. The main symptoms were downward rolling and reddening of the leaves (Figure 1a) in red varieties and yellowing of leaves in white varieties. In surveys performed in the year 2000, the total number of positive grapevine plants to these phytoplasmas was 11 out of 94 [29]. In the analysis carried out in the following years, these phytoplasmas were not detected again in grapevine plants.

### 2.4. Colombia

In Colombia, oil palm lethal wilt (OPLW) affects oil palm plantations, and the infected plants exhibit symptoms that resemble those associated with FY in Brazil. The phytoplasmas in diseased oil palm plants were identified as ‘*Ca*. P. asteris’, ribosomal subgroup 16SrI-B [30]. Moreover, collective RFLP characterization of the *groEl*, *amp,* and *rp* genes, together with sequence data, distinguished the strain detected in Colombian oil-palm samples from other aster yellows phytoplasmas used as reference strains—in particular, from an aster yellows strain infecting corn in the same country and associated with reddening symptoms (Figure 1b,c) [30]. Reports in Bogota areas of ‘*Ca*. P. asteris’ affecting woody hosts such as *Liquidambar styraciflua* L., *Fraxinus uhdei* (Wenz.) Lingelsh., *Populus nigra* L., *Pittosporum undulatum* Vent., and *Croton* spp., showing generic decline symptoms in single infection or in mixed infections, are also available in this Country [31,32].

### 2.5. Ecuador

In a recent epidemic of the potato purple top (PPT) [33] disease, symptomatic potato plant samples tested positive for phytoplasmas enclosed in the ribosomal subgroup 16SrI-F (‘*Ca*. P. asteris’), for the first time detected in potato and on the American continent. Diseased samples were collected from different locations and the phytoplasma presence was detected in diverse portions of the plants. The sequences of three PPT phytoplasma strains were deposited in GenBank under acc. nos. MG272306, MG272307 and MG272308 [33].

### 2.6. Paraguay

A phytoplasma affiliated with group 16SrI was identified in association with sesame (*Sesamum indicum* L.) [34], displaying symptoms of phyllody, virescence, mild leaf chlorosis, intense shoot proliferation, and the production of numerous small, deformed leaves. RFLP and phylogenetic analysis revealed that this phytoplasma belongs to subgroup 16SrI-B. The sequence designated as SePhy-Br01 was deposited in GenBank under acc. no. KY933669. Sesame is a new host species of 16SrI-B phytoplasma in Latin America [34]. Maize bushy stunt disease is a concern in Paraguay, and the research is concentrated on the assessment of maize cultivars and hybrids becoming infected during the main and the second crop [35]. Moreover, samples from plants infected with cassava frogskin disease showed the presence of group 16SrI phytoplasmas in 75% of the tested samples [36].

### 2.7. Peru

Surveys indicated that phytoplasma-associated symptoms were present in both cultivated crops, including alfalfa, carrots, coconut, clover, maize, papaya, native potatoes, improved potato, tomato, oats, papaya, and coconut, as well as in other plants such as dandelion and *Catharanthus roseus* (L.) G. Don. Phylogenetic analysis of the sequences confirmed that most of the strains belong to the aster yellows group (16SrI) [37]. Corn plants showing symptoms of midrib chlorosis, leaf reddening, short internodes, ear proliferation, and plant growth reduction collected from nine localities in the provinces of Huancayo, Chupaca, and Jauja in the Junín region resulted infected by diverse phytoplasmas after amplification of the phytoplasma ribosomal 16S and ribosomal protein genes. The sequencing of these amplicons indicated the presence of ‘*Ca.* P. asteris’ and ‘*Ca.* P. pruni’-related strains, in some cases in mixed infections. The first phytoplasma shares 100% identity with the ‘*Ca.* P. asteris’ strains from maize [38].

## 3. ‘*Ca*. P. aurantifolia = citri’ and ‘*Ca*. P. australasiae = australasiaticum’

The distribution of phytoplasmas in group 16SrII was considered restricted to the Southeast Asian region for a long time; then, recently diseases associated with phytoplasmas in some 16SrII subgroups have started to be discovered also in South America. In particular, the previously described ‘*Ca*. P. aurantifolia’ [2] and ‘*Ca*. P. australasiae’ [2] were those detected that were recently renamed as ‘*Ca*. P. citri’ and ‘*Ca*. P. australasiaticum’, respectively [39].

### 3.1. Bolivia

A phytoplasma was detected in peach trees showing symptoms very similar to those of peach yellow leaf roll [40]; however, it was wrongly classified. Revising the deposited sequence from peach in Bolivia (GenBank acc. no. AY725212), it has 99% identity with several strains belonging to the ribosomal subgroup 16SrII-D and with the strain having GenBank acc. no. Y10095; therefore, it is a ‘*Ca.* P. australasiae = australasiaticum’ strain [2,39]. Moreover, strains of ‘*Ca*. P. aurantifolia = citri’ [39] were detected in plants of *Podocarpus macrophyllus* Kusamaki with shortened internodes, leaf size reduction and proliferation; in rose, showing little leaf and yellowing; and in tomatillo (*Physalis ixocarpa* Brot. Ex Hornem.), with leaf deformation, crinkling, and curling [41].

### 3.2. Brazil

A work by Barros et al. [42] recorded, for the first time, the presence of 16SrII phytoplasmas in Brazil. *C. roseus* with witches’ broom symptoms from the states of São Paulo and Rio Grande do Norte were infected by 16SrII phytoplasmas identified on the basis of RFLP analysis. Another report of 16SrII phytoplasmas in *C. roseus* from São Paulo state, identified through RFLP, with three endonucleases was published [43]. The presence of phytoplasma strains closely related to ‘*Ca*. P. aurantifolia = citri’ was detected in the *Tabebuia pentaphylla* = *rosea* (Bertol.) DC. witches’ broom disease (GenBank acc. no. EF647744) [44] and in *Eucalyptus urophylla* S.T. Blake showing witches’ broom and little leaf symptoms (GenBank acc. no. KM597065) [45].

It is well known that citrus-associated phytoplasmas induce various symptoms that are either specific (witches’ brooms) or nonspecific (blotchy mottle leaves, lopsided fruits, reduced flowering, stunting, dieback, and decline). Interestingly, Silva et al. [46] assessed the presence of phytoplasmas in asymptomatic *Citrus* × *aurantifolia* (Christm.) Swingle (acid lime) from plants in the states of Minas Gerais, Santa Catarina, and São Paulo. The authors have obtained the first report of 16SrII-C phytoplasmas associated with *C. aurantifolia* in Brazil and the first report of asymptomatic citrus plants infected with a phytoplasma. In addition to Brazil, asymptomatic infections in lime trees were detected in Oman where infected trees with no symptoms collapsed in the fifth year after phytoplasma detection, making this asymptomatic variant pathogen potentially even more of a threat to global lime production [47].

### 3.3. Ecuador

In 2013, in the northern potato production region of Ecuador, symptoms of potato purple top disease (PPT) appeared in the fields on scattered plants. Characteristic symptomatology of this disease such as yellow and purple coloration of the upper leaflets, apical leaf curling, axillary buds, aerial tubers, and early senescence start to appear after flowering. In 2015, ‘*Ca*. P. aurantifolia = citri’ (16SrII group) deposited in GenBank under acc. nos. KT312845 and KT312846 was reported associated with PPT in Ecuador [48], and the finding was later confirmed in potatoes from tubers and inflorescences collected in the areas of Pichincha and Cañar [49].

### 3.4. Peru

One phytoplasma strain from potato was identified as ‘*Ca*. P. australasiae = australasiaticum’, belonging to the 16SrII group [37]. Symptomatic samples collected in Huayao exhibited yellowing of leaves, stunting, and little leaf. The 16S rRNA gene sequence was deposited in GenBank under acc. no. EU350562.

## 4. ‘*Ca*. P. pruni’

Phytoplasmas affiliated with the 16SrIII group are prevalent in South America and have been identified in various plant host species. Within group 16SrIII, several subgroups have been identified infecting distinct hosts, such as weeds, vegetables, fruit trees, ornamental plants, and staple crops. The most widespread phytoplasmas belong to the 16SrIII-J ribosomal subgroup [38]. Phytoplasmas in other ribosomal subgroups have been detected with lower prevalence, and they were fully or partially identified and characterized, in several South American countries.

### 4.1. Argentina

Phytoplasmas in the 16SrIII-B subgroup were detected in China tree, peach, plum, tomato, *Caesalpinia gilliesii* (Poinciana), and *C. roseus* plants. China tree plants showed branches with size reduction, leaf yellowing, and witches’ broom. Over the years, the symptoms worsened until the death of the plants [50]. Peach plants showed reddish and curled leaves, with necrotic leaf areas; there was also early defoliation and plant death [51]. Plum plants infected with this phytoplasma showed yellowing and witches’ broom symptoms [52]. Tomato, *C. gilliesii*, and *C. roseus* plants showed leaf size reduction and internode shortening [53].

The 16SrIII-J ribosomal subgroup infects several plant species: garlic, tomato, summer squash, *Bellis perennis* L., sunflower, cassava, and sugar beet. The symptoms of infected garlic plants were leaf reddening or yellowing, decline, and plant death. Bulb production was low, and cloves were deformed [50]. Tomato plants showed reddish and coriaceous leaves, and in summer squash and *B. perennis,* virescence symptoms appeared [53]. Sunflower plants showed virescence, phyllody, flower malformation, shortened internodes, and abnormal branches [54]. Dwarf cassava plants with witches’ broom and chlorosis were infected with the 16SrIII-J phytoplasma [55]. Wilting and yellowing were observed in sugar beet, fodder beet, and chard plants infected with the same phytoplasma [56].

The ribosomal subgroup 16SrIII-W was found in *Heterothalamus alienus* (Spreng.) Kuntze with leaf size reduction. The 16SrIII-X phytoplasma was detected in *E. bonariensis* plants with flower bud proliferation and lettuce plants showing witches’ broom, flowering malformation, and reddish leaves [53,57].

### 4.2. Bolivia

Yellows symptoms observed in China tree plants have been associated with phytoplasma 16SrIII-J [58]. Not fully characterized phytoplasmas belonging to the 16SrIII ribosomal group have been reported in bell pepper, strawberry, *Schinus molle* L., and *Arracacia xanthorrhiza* Bancr. The symptoms observed in plants were leaf size reduction and shortening of internodes in bell pepper; rosette and small fruits in strawberry; witches’ broom and yellowing in *S. molle*; yellowing and little leaf in *A. xanthorrhiza* [59].

### 4.3. Brazil

Barros et al. [42] are among the first who characterized phytoplasmas belonging to group 16SrIII. They investigated the presence of these pathogens in association with diseases in cassava (witches’ broom), eggplant (giant calyx and witches’ broom), sun hemp (witches’ broom), and maize bushy stunt. The identity of the phytoplasmas that were affiliated with subgroup 16SrIII-B was demonstrated through RFLP analysis with a few restriction enzymes. Several years later, Flôres et al. [60] reported the sequencing of the 16Sr RNA gene of phytoplasmas associated with cassava witches’ broom (CWB) disease in fields located in the state of São Paulo. Sequence analysis revealed that the phytoplasma strains cluster with members of the 16SrIII-B subgroup. Selected sequences were deposited in GenBank (CaWB-Br01, acc. no. GU193976; and CaWM-Br02, acc. no. GU193977). In a cassava field, the 16SrIII-B phytoplasma (GenBank acc. no. JX028239) was also identified in the weed *Leonurus sibiricus* Zamnesia, displaying small, shriveled, and chlorotic leaves: however, whether the cassava plants were phytoplasma infected was not mentioned [61].

Other 16SrIII phytoplasmas are associated with cassava frogskin disease (CFSD), which mainly affects the tubers (roots), while the aboveground organs are asymptomatic. In 2014, phytoplasmas belonging to subgroup 16SrIII-L (GenBank acc. nos. KF019184 and KF019185) were found in association with typical symptoms of CFSD in genotypes maintained in areas of the Brazilian Cassava Germplasm (Embrapa Cassava & Fruits, Cruz das Almas, Bahia state) [62]. In the same year, a phytoplasma affiliated with group 16SrIII, subgroup A, was found in cassava plants with CFSD from the Minas Gerais state. The etiology of the disorder has been controversial among researchers worldwide; some of them believe the causal agent is a virus, while some of them attribute the disease to phytoplasmas. Interestingly, after the samples were assessed for the presence or absence of the virus and phytoplasma, both agents were found in diseased tissues, and the authors reported the co-infection of the cassava plants with the dsDNA virus and the 16SrIII-A phytoplasma [63].

Eggplants displaying symptoms of giant calyx can harbor distinct 16SrIII subgroups. Barros et al. [42] found the 16SrIII phytoplasma associated with giant calyx disease of eggplant, and the strain was characterized as subgroup 16SrIII-B. Years later, virtual RFLP and phylogenetic analysis enabled the classification of subgroups 16SrIII-J and 16SrIII-U of phytoplasmas from plants cultivated in two municipalities in São Paulo state. Notably, the 16SrIII-J phytoplasma (GenBank acc. no. HM589212) was identified in plants grown in Piracicaba, and the subgroup 16SrIII-U was detected in plants cultivated in Bragança Paulista, São Paulo state [64]. In plants from a commercial field in the vicinity of Bragança Paulista, tomato big bud disease, characterized by calyx deformation among other symptoms, was described. A sequence of the 16S rRNA gene of the phytoplasma (TBB-Br-A; GenBank acc. no. AY863192) revealed its affiliation with group 16SrIII, but the phytoplasma subgroup was not identified [65].

It is remarkable that in Brazil, sweet orange trees with symptoms of “huanglongbing” (HLB) were found infected by phytoplasmas despite testing free for ‘*Candidatus* Liberibacter asiaticus’ and ‘*Ca*. L. americanus’. This finding was reported by Teixeira et al. [66] and a 16SrIX phytoplasma—named HLB-associated phytoplasma—was identified in the symptomatic plants. More recently, samples with symptoms of HLB from the states of São Paulo and Minas Gerais tested free for the presence of the bacteria and phytoplasma 16SrIX but were infected with phytoplasmas belonging to group 16SrIII (unspecified subgroup) and to two subgroups, 16SrIII-B and 16SrIII-X [67]. The role of weeds as a source of inoculum to the citrus phytoplasmas needs to be further investigated to understand the disease epidemiology and to design appropriate disease management strategies. In citrus orchards infected with the 16SrIII-X phytoplasma in the state of São Paulo, plants of beggarticks (*Bidens pilosa* L.) with virescence and phyllody, and honey weed (*L. sibiricus*) exhibiting leaf distortion were harboring phytoplasmas of the same subgroup [68].

Brazilian species native to the “cerrado” area, *Vernonia brasiliana* (L.) Druce, exhibiting shoot proliferation was observed in grasslands in the municipalities of Patos de Minas and Piracicaba, respectively located in the states of Minas Gerais and São Paulo. In the symptomatic plants, a phytoplasma of the 16SrIII-B subgroup was identified (strain VbSP-Br12, GenBank acc. no. KX273432). In addition to the diseased plants, asymptomatic *V. brasiliana* was collected from pasture fields of Patos de Minas and Piracicaba and of a third municipality, Maringá (Paraná state). Surprisingly, the 16SrIII-B phytoplasma was identified in asymptomatic samples from Maringá, and the 16SrIII-J phytoplasma (strain VbSP-Br44, GenBank acc. no. KX273433) was found in asymptomatic *V. brasiliana* plants of Minas Gerais [69]. In the wake of distinct phytoplasmas recorded in a single plant host, the weed known as field mustard (*Brassica rapa* L.) can harbor three subgroups within group 16SrIII, in addition to subgroup 16SrVII-B. Diseased plants, widely growing in cauliflower commercial fields in São Paulo state, showed intense proliferation of thin branches and small and deformed leaves and flowers. The authors reported the simultaneous presence of phytoplasmas in subgroups 16SrIII-B, 16SrIII-J, and 16SrIII-U [70].

Passion fruit (*Passiflora edulis* f. *flavicarpa* Sims) is an important fruit in Brazil that has been known to be affected by passion fruit witches’ broom disease since the 1980s [71]. Symptoms of the disease comprise generalized chlorosis, short internodes, shoot proliferation, and small and coriaceous leaves. In 2012, Davis et al. [72] published a new subgroup 16SrIII-V in diseased passion fruit from Bonito, Pernambuco state (strain PassWB-Br4, GenBank acc. no. GU292082).

The diversity of 16SrIII phytoplasma subgroups reported in Brazil has been expanding in recent years. According to Ferreira et al. [73], a 16SrIII-F phytoplasma was identified in “acerola” (*Malpighia emarginata* DC) with symptoms of shoot proliferation. It was the first identification of this ribosomal subgroup, described in USA only, and strain ASP-Br01 was deposited in GenBank under acc. no. MT153591. Plants in commercial fields of chrysanthemum (*Chrysanthemum morifolium* Ramat) in São Paulo state displayed color breaking and virescence, in which the 16SrIII-X *Chrysanthemum* color-breaking phytoplasma (strain ChViCb-Br01, GenBank acc. no. MN535228) was identified [74].

**Figure 2:**
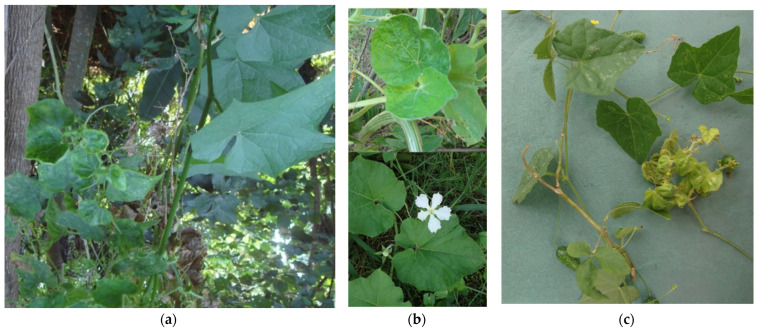
Plant species infected with ‘*Ca.* P. pruni’ strains in Brazil: in (**a**) chayote with witches’ broom (16SrIII-J); in (**b**) *Lagenaria siceraria,* with reduced leaf size (16SrIII-Y) (top) and asymptomatic (bottom); and in (**c**) *Melothria pendula*, showing leaf malformation (16SrIII-U).

Despite the variety of phytoplasma subgroups in group 16SrIII, subgroup 16SrIII-J is prevalent in Brazil. It was described for the first time in association with chayote witches’ broom (ChWB) disease (Figure 2a), in samples from Rio de Janeiro where the disease was first observed in the 1960s. Vegetating nearby chayote fields, symptomatic bitter gourd (*Momordica charantia* L.) showing witches’ broom was also infected with 16SrIII-J and it is a potential alternative host of the phytoplasma since it grows wild and widely [75]. A phytoplasma strain member of subgroup 16SrIII-B was reported in bitter gourd from the state of Minas Gerais [76]. In addition to chayote and bitter gourd, other members of the Cucurbitaceae family can be infected by phytoplasma 16SrIII-J. This phytoplasma was found in association with pumpkin (*Cucurbita moschata* Duchesne ex Poir) yellows in the fields next to chayote crops [77]. The phytoplasma associated with loofah witches’ broom disease from Rio de Janeiro state was identified as belonging to subgroup 16SrIII-J [78] as well as the phytoplasma in *Sicana odorifera* (Vell.) Naudin with witches’ broom disease [79]. The predominance of the 16SrIII phytoplasma group in the cucurbits is remarkable. A phytoplasma assigned to the new subgroup 16SrIII-Y was found in bottle gourd (*Lagenaria siceraria* L.) with reduced leaf size, leaf malformation, and yellowing (Figure 2b) from the Rio de Janeiro state [80], while diseased Guadeloupe cucumber (*Melothria pendula* L.) (Figure 2c) was a host for a strain of subgroup 16SrIII-U (GenBank acc. no. MK108032) [81].

Stunt disease of varieties of *Brassica oleracea* L. was identified in association with phytoplasmas of the 16SrIII group in municipalities of the state of São Paulo. The disease is characterized by plant stunting, inflorescence malformation, leaf reddening, and phloem necrosis and was found in association with distinct phytoplasma groups. Cabbage (*B. oleracea* var. *capitata*) stunt is associated with a 16SrIII phytoplasma, but no subgroup has been attributed to the pathogen [22]. Cauliflower (*B. oleracea* var. *botrytis*) stunt phytoplasma was characterized as subgroup 16SrIII-J, and its sequence, designated as CfS, was deposited in GenBank under acc. no. HM237045 [82]. Stunt disease of broccoli (*B. oleracea* var*. italica*) was associated with an unidentified subgroup of 16SrIII in the first report of the disorder. The phytoplasma strains were designated as BSP21, BSP73, and BSP76 and the sequences were deposited in GenBank under acc. nos. JX626327, JX626332, and JX626331, respectively [20]. In a further study, broccoli stunt phytoplasma strain BSP21 was classified as the representative of the new subgroup Z in group 16SrIII [21]. Years later, during the investigation for phytoplasma insect vectors, a phytoplasma affiliated with subgroup 16SrIII-X (GenBank acc. no. MG988412 and MG988413) was found in diseased broccoli, and the leafhoppers *Agallia albidula* (Uhler 1895) (Cicadellidae; Agalliinae*)*, *Agalliana sticticollis* (Stal 1859) (Cicadellidae; Megophthalminae), *Atanus nitidus* (Linnavuori 1955) (Cicadellidae; Deltocephalinae*)* and *Balclutha hebe* (Kirkaldy 1906) (Cicadellidae; Deltocephalinae*)* were identified as potential vectors of this broccoli stunt phytoplasma strain [83].

Phytoplasmas of subgroup 16SrIII-J were found in samples of the ornamental species *Celosia argentea* L. and *C. spicata* L. from the state of São Paulo. The symptoms exhibited by the two species comprised leaf malformation, shoot proliferation, and stunt. As at the molecular level, the strains were indistinguishable from each other, the consensus sequence designated as CelLM4/Br1 was deposited in GenBank under acc. no. JN574433) [84]. A disease named *Aegiphila* witches’ broom was described in *Aegiphila verticillate* Vell., a species native to the Brazilian “cerrado”. Plants displaying symptoms of intense proliferation and small and slightly chlorotic leaves were observed in Divinópolis, Minas Gerais state. In addition, the premature fall of flowers and small fruits was recorded. The phytoplasma was designated as strain AegWB-Br1 (GenBank acc. no. KT148597) and it is affiliated with subgroup 16SrIII-J [85]. Fugita et al. [69] reported *Brachiaria decumbens* Stapf. as a symptomless host of a 16SrIII-J phytoplasma designated *Brachiaria*–Brazil 01 phytoplasma (Brach-Br01; GenBank acc. no. KX342017). China tree or Chinaberry tree (*Melia azedarach* L.) is a species with multiple applications, including its use as an ornamental and a shade tree in Southern Brazil where the presence of the disease named decline of China tree has been restraining its use. The decline of the China tree is associated with a 16SrIII-B phytoplasma (GenBank acc. no. FJ404775) [86]. Bougainvillea (*Bougainvillea spectabilis* Willd.) potted plants exhibiting shoot proliferation symptoms were observed in commercial nurseries in São Paulo state. RFLP and phylogenetic analysis enabled the identification of 16SrIII-B phytoplasmas in the diseased plants [19].

In Brazil, there are records of 16SrIII phytoplasmas infecting different plant hosts, but the subgroups were not defined by the time the reports were released. Begonia shoot proliferation phytoplasma was identified in nested PCR with specific primers [87]. Molecular identification using group-specific primer pair and RFLP analysis revealed the presence of phytoplasmas of group 16SrIII in commercial poinsettia [88], apple rubbery wood [89], strawberry phyllody [25], and summer squash yellows [90].

### 4.4. Chile

**Figure 3:**
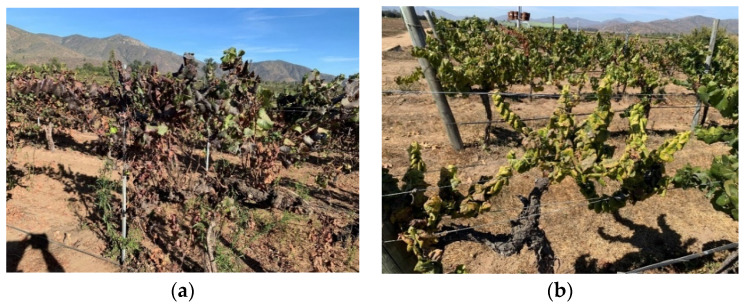
Grapevine yellows in Chile: (**a**) variety Pinot noir with reddening of the leaves; (**b**) variety Chardonnay with yellowing of the leaves.

Several plant species were infected by the 16SrIII-J phytoplasmas—such as potato, prickly pear, carrot, lettuce, Swiss chard, sugar beet, cherry, and grapevine. The symptoms observed were as follows: leaf curl, witches’ broom, and yellowing in potato plants; phloem necrosis and bud deformation in prickly pear; reddening of the leaves, deformation of the floral buds, and proliferation of lateral roots in carrot; sprout proliferation and leaf deformation in lettuce; general yellows, stunting, and distorted leaves in Swiss chard; rolled-up leaves with necrosis of the edge and yellowing in sugar beet; a decay, low vigor, and even death in cherry plants; downward leaf rolling and yellowing or reddening of the leaves in white and red grapevine varieties, respectively [91,92,93,94]. Epidemiological studies on grapevine yellows (GY) (Figure 3) have verified the presence of seven 16SrIII-J phytoplasma reservoir species: *Convolvulus arvensis* L., *Galega officinalis* L., *Polygonum aviculare* L., *Rosa* spp., *Brassica rapa* L., *Erodium* spp., *Malva* spp., and *Rubus ulmifolius* Schott. Two leafhopper species, *Paratanus exitiosus* Beamer 1943 (Cicadellidae; Deltocephalinae) and *Bergallia valdiviana* Berg 1881 (Cicadellidae; Megophthalminae), have been demonstrated to be vectors of the 16SrIII-J phytoplasma. In addition, five other leafhopper species have been identified as potential vectors: *Amplicephalus ornatus* (Cicadellidae; Deltocephalinae) Linnavuori 1959, *A. pallidus*, *A. curtulus*, *Bergallia* sp., and *Exitianus obscurinervis* Stal 1959 (Cicadellidae; Deltocephalinae). These seven leafhoppers feed on weeds and only occasionally on grapevine, allowing the transmission of the phytoplasmas to this species [94,95]. The wide presence of these insect vectors and weed species would explain the high dissemination of 16SrIII-J phytoplasma in a large number of crops in Chile. The first draft genome sequence of the 16SrIII-J phytoplasma has been obtained (GenBank acc. no. LLKK00000000). Based on this achievement, 16SrIII-J phytoplasma SAP54 and SAP05 orthologous genes have been identified as responsible for flowering abnormalities in *Nicotiana benthamiana* Domin and *Arabidoposis thaliana* (L.) Heynh plants inoculated with *Tobacco mosaic virus* vector carrying the genes [96].

### 4.5. Colombia

**Figure 4:**
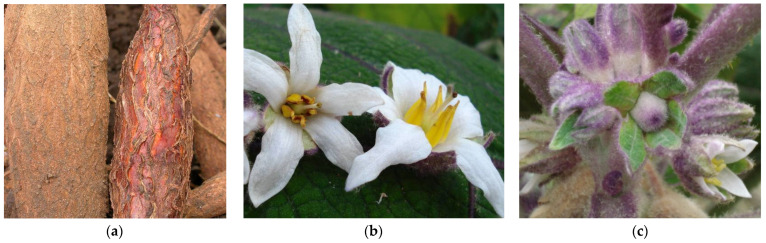
Symptoms of ‘*Ca*. P. pruni’ in Colombia: (**a**) cassava roots in which the one on the right is infected by 16SrIII-L phytoplasmas; (**b**,**c**) *S. quitoense* (“lulo”) with flower and bud malformations infected by 16SrIII-F phytoplasmas.

Cassava frogskin disease (CFSD) is an economically important root disease of cassava (*Manihot esculenta* Crantz) in Colombia and other South American countries. The roots of severely affected plants are thin, making them unsuitable for consumption also due to the presence of thick peel (Figure 4a). Phytoplasma infections were confirmed in 35 cassava genotypes exhibiting mild or severe CFSD symptoms by the identification of group 16SrIII strains using RFLP and sequence analyses. CFSD strains were assigned to ribosomal and ribosomal protein subgroups 16SrIII-L and rpIII-H, respectively [97]. Phytoplasmas of the 16SrIII-F subgroup were identified in samples of *Solanum quitoense* Lam (“lulo”) (Figure 4b,c) and *Physalis peruviana* L. (“uchuva”), showing severe malformations in the reproductive structures, and were further characterized on *tuf* and *rp* genes that confirmed their genetic identity [98]. A phytoplasma (strain CDD, GenBank acc. no. AY525125) affiliated with the 16SrIII group was found in association with coffee crispness disease, locally known as “crespera”. It is characterized by affecting the aerial parts of the plant, especially the leaves, floral buds, and berries, causing leaf proliferation and phyllody. The CDD phytoplasma is the first reported phytoplasma in the genus *Coffea* [99].

### 4.6. Paraguay

In Paraguay, cassava frogskin disease (CFSD) is associated with a phytoplasma affiliated with subgroup 16SrIII-L [100] as the phytoplasma found in CFSD in Colombia [97]. The frogskin phytoplasma from Paraguay was identified using PCR with group-specific primer pairs and sequencing. Sequences from the frogskin phytoplasma were deposited in GenBank under acc. nos. KF701485, KF701497, and KF701498. China tree (*M. azedarach*) is a species adopted in Paraguay and in several countries in South America where it is affected by phytoplasma diseases reported in Argentina, Bolivia, Brazil, and Paraguay. Diseased China trees exhibiting symptoms of decline, yellowing, and little leaf were observed in the cities of Caazapá, Villarrica, Itaguá, and Asunción. Phytoplasma identification revealed the presence of 16SrIII and 16SrXIII phytoplasmas in single and mixed infections. The phytoplasmas of both groups were found in one sample collected in Itaguá and in a sample from Asunción. RFLP patterns identical to subgroup 16SrIII-B were found for the phytoplasma detected in the tree sampled in Itaguá [101].

### 4.7. Peru

Faba bean samples with symptoms of yellowing, dwarfism, shoot proliferation, inter-node shortening, leaf sprouts, and lack of pod and seed production from Huancayo and Chupaca provinces, Junin, were analyzed to verify phytoplasma presence and identity. The amplification on the 16S ribosomal gene followed by restriction fragment length polymorphism and sequence analysis allowed for the classification of the detected phytoplasma in subgroup 16SrIII-J. The phytoplasma identity was also verified by the amplification on the ribosomal protein gene amplicons obtained with primers specific for the phytoplasmas enclosed in the 16SrIII group; it is the first description of a disease associated with phytoplasmas in faba beans in the country [102]. Corn plants showing symptoms of midrib chlorosis, leaf reddening, short internodes, ear proliferation, and plant growth reduction showed in a few cases of a mixed infection of ‘*Ca*. P. asteris’ (16SrI-B) and ‘*Ca.* P. pruni’ (16SrIII-J) strains. The latter showed 99.82% identity with ‘*Ca*. P. pruni’. This is the first report of the 16SrIII-J phytoplasma associated with maize bushy stunt disease [38]. Furthermore, group 16SrIII phytoplasmas were reported in tomato from Ica (GenBank acc. no. EU882813) and in dandelion *Taraxacum officinale* (Weber) ex Wiggers] from Macartuna (GenBank acc. no. EU350563), which was the first report of a 16SrIII phytoplasma in dandelion on the American continent [37].

## 5. *‘Ca.* P. ulmi’ and ‘*Ca*. P. ziziphi’

### 5.1. Brazil

Sunn hemp (*C. juncea*) is a host of phytoplasmas affiliated with distinct 16Sr groups and subgroups. In 2004, plants exhibiting reduced and chlorotic leaves, shortening of internodes, and shoot proliferation were observed in São Paulo state. A phytoplasma belonging to subgroup 16SrV-B (‘*Ca*. P. ziziphi’) was identified in the symptomatic samples on the basis of nested PCR using group-specific primers and RFLP analysis [103].

### 5.2. Chile

‘*Ca.* P. ulmi’ (16SrV-A) was detected in grapevine plants with GY symptoms; in orange, lemon, and mandarin plants, showing leaf yellowing; and in “murtilla” (*Ugni molinae* Turcz.), a spontaneous bushy plant belonging to the Myrtaceae family growing in the south of Chile, exhibiting witches’ broom. It has been demonstrated that the leafhopper *A. curtulus* transmitted ‘*Ca.* P. ulmi’ from *U. molinae* to ryegrasses (*Lolium multiflorum* cv. *Tama*). Furthermore, transovarial transmission of ‘*Ca.* P. ulmi’ has not been observed in *A. curtulus* but it was also detected in *Citrus* plants [92,104,105].

### 5.3. Colombia

At the end of 2010, severe disease was observed in *S. tuberosum* in the variety Criolla Colombiana. The main symptomatology consisted of discoloration or yellowing of the whole plant, apical leafroll, dwarfing, axillary buds, and thicker internodes. Phytoplasmas related to 16SrV and 16SrXII groups were identified by nested PCR assays followed by real and virtual RFLP and sequence analyses. This was the first report of phytoplasma presence in this species in Colombia and the first identification of group 16SrV phytoplasmas in potatoes [106]. In samples from 21 liquidambar trees from Bogota, nested PCR assays identified phytoplasmas belonging to the16SrV-B (‘*Ca*. P. ziziphi’) in single or mixed infections [31].

### 5.4. Ecuador

Phytoplasmas identified as ‘*Ca.* P. ulmi’ were detected in a small number of samples of potato showing purple top symptoms collected in Pichincha in a mixed infection with 16SrII phytoplasmas [49].

## 6. ‘*Ca*. P. sudamericanum’

### Brazil

Passion fruit witches’ broom disease was first reported in the states of Rio de Janeiro and Pernambuco by Kitajima et al. [71]. After several decades, the disease was reported in the states of Bahia, Paraná, Sergipe, and São Paulo, and its association with phytoplasmas was demonstrated by PCR analysis [107]. The taxon ‘*Ca*. P. sudamericanum’ was proposed to be designated as an undescribed phytoplasma associated with plants of passion fruit with witches’ broom disease. Infected plants were found in the state of Minas Gerais exhibiting symptoms of abnormal proliferation of axillary shoots and witches’ broom growths. The strain PassWB-Br3 is the reference strain of the described ‘*Candidatus*’ taxon (GenBank acc. no. GU292081) and is representative of subgroup 16SrVI-I [72]. To present knowledge the ‘*Ca.* P. sudamericanum’ has not been reported elsewhere.

## 7. ‘*Ca*. P. fraxini’

The geographic origin of ‘*Ca*. P. fraxini’ is very likely on the American continent [31,32]; however, there are sporadic reports of its presence in Europe and Asia.

### 7.1. Argentina

‘*Ca.* P. fraxini’ (16SrVII-C) subgroup was described in alfalfa plants with witches’ broom and strawberry plants showing phyllody symptoms [108,109,110]. *Artemisia annua* L. and *E. bonariensis* with yellowing and witches’ broom symptoms were infected with 16SrVII-B phytoplasmas [111]. The phytoplasma detected in artemisia was named *Artemisia* witches’ broom (ArtWB). The sequences of three strains were deposited in GenBank: ArtWB-I (acc. no. DQ989178), ArtWB-II (acc. no. DQ989179), and ArtWB-III (acc. no. DQ989180).

### 7.2. Brazil

**Figure 5:**
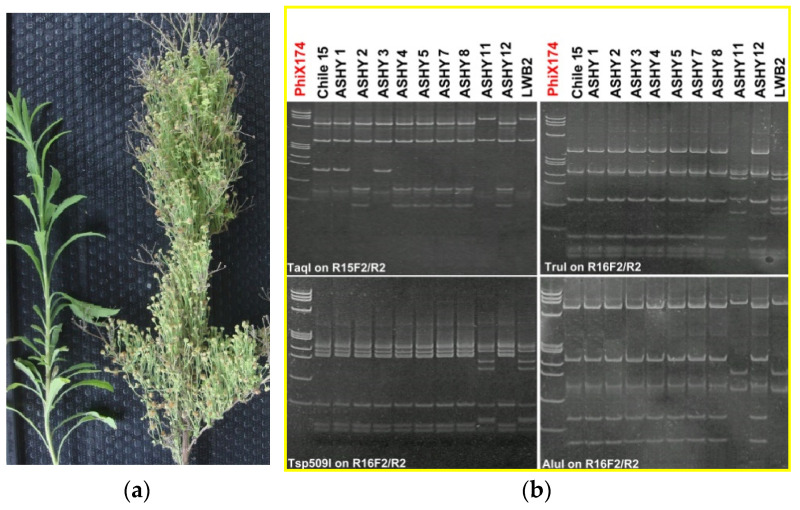
Erigeron showing severe witches’ broom symptoms associated with the presence of ‘*Ca.* P. fraxini’ (16SrVII) in (**a**) on the right; in (**b**) polyacrylamide 6.7% gels showing the restriction profiles of phytoplasma 16SrVII-A from Chile (Chile 15) compared with several strains of ash yellows (ASHY 1 to 8 and 11, 12, and LWB2) from the United States of America, kindly provided by W. Sinclair. The enzymes are at the bottom of each figure and the marker (PhiX174) is *Hind*III digested.

The first report of a representative of the ash yellows phytoplasma group outside North America emerged from South America, Brazil. This phytoplasma was found in naturally diseased plants of *Erigeron* sp. (Figure 5a) and periwinkle in the state of São Paulo. Plants exhibited a reduced size of leaves, chlorosis, and proliferation of axillary shoots resulting in prominent witches’ broom growths. The phytoplasma strains detected in erigeron and periwinkle were respectively designated as *Erigeron* witches’ broom (EriWB; GenBank acc. no. AY034608) and Rio das Pedras witches’ broom (RPWB; GenBank acc. no. AF411592). EriWB and RPWB did not show sequencing divergence, and both are members of subgroup 16SrVII-B [112]. Several years later in the Rio de Janeiro state, phytoplasmas affiliated with the same subgroup (16SrVII-B) were detected in witches’ broom-diseased *E. bonariensis*, indicating that weeds of the genus *Erigeron* can host phytoplasmas broadly. Sequences of this strain (ErB8) from Rio de Janeiro were submitted to GenBank under acc. nos. KP202353 (16S rRNA gene) and KP202354 (*tuf* gene). In 2016, naturally diseased plants of ming aralia [*Polyscias fruticose* (L.) Harms] and cauliflower were found in the state of São Paulo. In ming aralia, the symptoms exhibited were yellowing and abnormally small leaves, and the strain MaLL Br01 (GenBank acc. no. KR3631280) was sequenced [113]. The subgroup 16SrVII-B phytoplasma was found in association with cauliflower stunt disease. Infected plants showed stunting, reddening of the leaves, malformed inflorescences, and vessel necrosis. The phytoplasma strain was designated CfS-Br10, and its sequence deposited in GenBank is under acc. no. KR270802 [114]. In areas of the green belt region of the city of São Paulo, with cauliflower plants showing stunt disease, the weed known as field mustard (*B. rapa*) was infected with a strain of a 16SrVII-B phytoplasma, the same subgroup identified in stunted cauliflower [70]. Disease associated with a 16SrVII-B phytoplasma was described in *P. peruviana* displaying leaf malformation, chlorosis, and shoot proliferation in São Paulo state. The phytoplasma sequence, designated as *Physalis* peruviana yellow (Ppy-Br01), was deposited in GenBank under acc. no. MT218429 [115].

In Brazil, olive is emerging as an important crop, and olive oil is a potential trade commodity. Phytoplasmas can pose a threat to olive expansion in the country. In 2015, commercial plantings located in the state of Minas Gerais exhibited a high incidence of symptomatic trees showing slow development, yellowing, shoot proliferation, small leaves, and yield reduction. The association of a 16SrVII-B phytoplasma and diseased plants have been demonstrated, and the disease was named olive witches’ broom. RFLP and phylogenetic analysis enabled the identification of the phytoplasma strain, designated as OWB-Br01 (GenBank acc. no. MH141985) [116]. A 16SrVII-C subgroup phytoplasma (strain CrSP-Br01; crotalaria shoot proliferation; GenBank acc. no. KC756947) was found in the state of São Paulo in association with shoot proliferation symptoms in *Crotalaria juncea* [117]. Subgroup 16SrVII-D was designated for the representative EboWB phytoplasma (*E. bonariensis* witches’ broom; GenBank acc. no. KJ831066). The phytoplasma was found in plants exhibiting witches’ broom observed in areas adjacent to a passion fruit orchard in the state of São Paulo [110]. Another representative of a new phytoplasma subgroup in the ash yellow group, 16SrVII-F, was described in *V. brasiliana*. This species belongs to the Asteraceae family and grows in pasture areas in several states. Diseased plants have been found with symptoms of shoot proliferation and deformed and chlorotic leaves in the fields in the states of Minas Gerais, Paraná and São Paulo. The representative strain of the subgroup 16SrVII-F was named VbSP-BR29, and its sequence was deposited in GenBank under acc. no. KX342018 [118]. A phytoplasma strain identified as CTb1-BR (GenBank acc. no. MT396670) was a representative strain of subgroup 16SrVII-G and it was identified in sweet orange (*C. sinensis*) trees in commercial orchards in the state of São Paulo. Citrus infected by the CTb1-BR strain exhibited chlorotic leaves and blotchy mottling, typically associated with HLB [119].

### 7.3. Chile

‘*Ca.* P. fraxini’ (16SrVII-A) strains were detected in grapevine, *U. molinae*, and *Paeonia lactiflora* Pall. [29,95,120]. In grapevine plants the symptoms observed were downward rolling of leaves, reddening of leaves in red varieties, and yellowing of leaves in white varieties. During the first survey, the number of positive grapevine plants was 13 out of 94. The strain infecting grapevines was undistinguishable after RFLP analyses on 16S rRNA gene amplicons with different restriction enzymes from the reference strain ASHY1, that is, ‘*Ca*. P. fraxini’ (Figure 5b). In a more recent survey, 2% of positive samples were found in the total of 330 grapevine plants analyzed. Witches’ brooms were present in *U. molinae* plants. *P. lactiflora* with malformation, necrosis, and downward rolling of leaves, green stripes on the petals, and drying-up of the flower bud, a result of being infected by 16SrVII-A phytoplasmas [29,95,120].

### 7.4. Colombia

‘*Ca.* P. fraxini’ (16SrVII-A) was reported to widely infect ash and liquidambar mainly in the city of Bogota [31,121]. Symptomatic liquidambar and ash plants showed symptoms of different intensity of yellowing and deformation of the tree crown, small leaves, tufted foliage, epicormic growth, and abnormal elongation of apical shoots.

## 8. ‘*Ca*. P. phoenicium’

### 8.1. Brazil

Phytoplasmas enclosed in the 16SrIX group are prevalent in the American continent citrus orchards. In 2008, a phytoplasma of group 16SrIX was identified by Teixeira et al. [66] in sweet orange trees showing blotchy mottle leaves and lopsided fruits carrying aborted seeds—symptoms indistinguishable from HLB. The 16SrIX citrus phytoplasma was designated as HLB-associated phytoplasma (GenBank acc. no. EU266074). Onward surveys in Brazilian citrus orchards revealed the presence of 16SrIX phytoplasmas in sweet orange in the states of Bahia [46], Minas Gerais [122], and in the Distrito Federal region [123]. After the discovery of phytoplasmas in citrus orchards, many surveys have been carried out to identify the presence of putative insect vectors and plants that can harbor the pathogen. In Brazilian citrus orchards, sunnhemp (*Crotalaria juncea*) is used as a cover crop. Phytoplasmas were detected in sunnhemp plants with witches’ broom and virescence collected between citrus rows or in the fields near orchards in São Paulo. The phytoplasma shares 100% identity with the HLB phytoplasma (group 16SrIX), and the finding suggests that sunn hemp is a major source of inoculum for the citrus plants [122]. Faunistic analysis carried out in São Paulo state indicated that leafhoppers within the genus *Scaphytopius* are potential vectors of the 16SrIX phytoplasma in citrus, and the species *S. marginelineatus* Stal 1859 (Cicadellidae; Deltocephalinae) harbor the phytoplasma [67,124]. In citrus orchards surveyed in Brazlandia, Distrito Federal, the weeds *B. pilosa*, *Euphorbia* sp. and *Sida* sp. were infected with the 16SrIX phytoplasma and could be the reservoirs for the citrus phytoplasma. In the same orchards, specimens of *Scaphytopius* sp. were found harboring the phytoplasma [125]. Periwinkle is another host to 16SrIX phytoplasmas in Brazil [126]. In a report from 1998, 16SrIX phytoplasmas were identified by RFLP analysis in diseased *C. roseus* showing witches’ broom symptoms from the states of São Paulo, Pernambuco, and Rio Grande do Norte [42]. The same phytoplasma group was identified in *C. roseus* plants displaying virescence in the state of Mato Grosso [43]. Phytoplasmas affiliated with group 16SrIX were identified in periwinkle plants exhibiting virescence, phyllody, and variegation; the plants were collected from public gardens in the municipalities of Carmo do Paranaíba (state of Minas Gerais) and Araraquara (state of São Paulo); two phytoplasma strains PwK-AR1 (GenBank acc. no. JN792515) and PwK-CP3 (GenBank acc. no. JN792516) shared 99% identity with ‘*Ca*. P. phoenicium’ [68].

### 8.2. Colombia

Phytoplasmas enclosed in this ribosomal group were repeatedly detected in periwinkle showing virescence and little leaf symptoms. RFLP analyses enclose Colombian strains in the 16SrIX-C subgroup. The 16SrIX group-specific primers provided amplification of *rp* gene, and RFLP analyses of these amplicons with *Tru*I and *Tsp509*I clearly distinguished the Colombian strains from the others. *Tuf* gene RFLP analyses performed with *Tru*I, *Hpa*II, and *Tsp509*I not only distinguished the Colombian strains from the others but also discriminated three strains from the other two [127]. Phytoplasmas in group 16SrIX were also identified in liquidambar trees showing decline symptoms but always in a mixed infection with phytoplasmas in other ribosomal groups [31].

## 9. ‘*Ca*. P. pyri’

It belongs to the 16SrX-C subgroup and is the most widespread phytoplasma in South America in pear and peach threes [128,129,130]. To date, it has not been possible to clarify the epidemiological cycle because of the two main known insect vectors, *Cacopsylla pyri* L. (Psyllidae) and *C. pyricola* Foerster 1848 (Psyllidae), the first one has never been found in South America, while the latter has only been found sporadically in Argentina.

### 9.1. Argentina

Peach plants showed chlorotic leaves, ridges, and thickening of the central veins. Subsequently the 16SrX-C phytoplasma was also detected in pear plants, which showed a remarkable decline with reddening of leaves and branch phloem necrosis [128,129].

### 9.2. Chile

**Figure 6:**
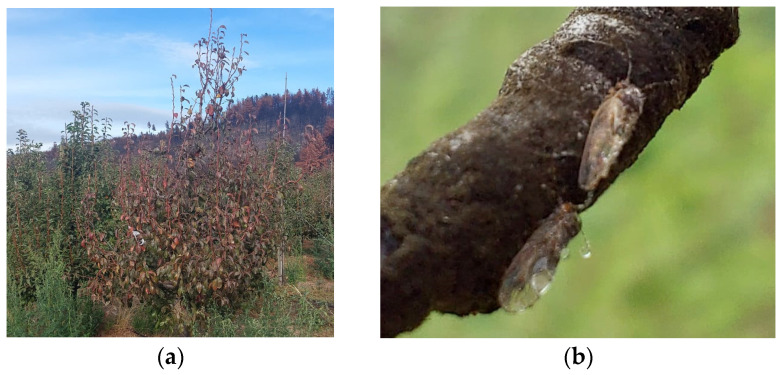
(**a**) Pear plant infected with ‘*Ca.* P. pyri’ with reddish leaves; (**b**) *C. bidens* individuals on a twig at the beginning of winter in Chile.

Pear plants with typical symptoms of ‘*Ca*. P. pyri’ (cv. Williams on quince BA29 rootstock) represent the first report of the pathogen presence in South America. The main symptom is the early reddening of the leaves in the season (Figure 6a) and the reduced fruit production. The prevalent psyllid in Chilean pear orchards is *Cacopsylla bidens* Sulc 1907 (Psyllidae) (Figure 6b), which has been repeatedly found positive for ‘*Ca*. P. pyri’. The transmission trials are ongoing to find out if it is a vector of the pathogen [130].

### 9.3. Uruguay

In Uruguay, the association of phytoplasmas with the disorder known as “decaimiento del peral” (pear decline) was confirmed in diseased pear orchards through PCR assessment although plants have been showing symptoms similar to pear decline since the 1990s [131]. The pathogen was found in psyllid specimens collected from diseased orchards and a positive correlation was observed between the incidence of the disease and the level of psyllid infestation [132]. Valle et al. [133] reported the presence of *C. bidens* throughout the pear production area in Uruguay, which has probably been misidentified as *C. pyricola* for years. The disease incidence is positively correlated with the psylla orchard infestation, but chemical control is not effective to prevent the transmission of the phytoplasma [134]. Emphasis has been dedicated to the investigation of the interaction between scion and rootstock to hamper the disease intensity since some of these combinations can exacerbate the disease symptoms [135].

## 10. ‘*Ca*. P. solani’

### 10.1. Brazil

So far, there is only one record of a 16SrXII group phytoplasma in Brazil. A strain affiliated with the “stolbur” group was identified through RFLP analysis in *Hibiscus rosa-sinensis* L. plants with witches’ broom symptoms. The phytoplasma was identified in samples collected during a survey to evaluate the epidemiological spreading of phytoplasmas diseases in *H. rosa-sinensis* [136].

### 10.2. Chile

‘Ca. P. solani’ was detected in peach and grapevine plants. The peach plants showed decay [137], while the grapevine plants had typical GY symptoms. The total number of positive grapevine plants was 15 out of 94 [29]. After these first surveys, the phytoplasma was never detected again in either plant species.

### 10.3. Colombia

In 2011 phytoplasma-related symptoms were found in potato for seed lots of multiplication in three areas in the municipalities of Guasca and Zipaquirá, (2900–3000 m above sea level), in the variety Criolla Colombiana. Symptoms of the disease are discoloration or yellowing of leaflets, apical leafroll, dwarfing, axillary buds, and thicker internodes. The 16SrXII-A phytoplasmas were always identified in a mixed infection with 16SrV phytoplasmas [106]. The ‘*Ca*. P. solani’ was detected alone and in mixed infection with other phytoplasmas in liquidambar trees in Bogota showing deformation of the crown, tufted foliage, deliquescent internodes, atypical purple pigmentation of leaves, abnormal elongation of apical shoots, small leaves, yellowing, purple patches in the crown, dead branches, defoliation, epicormics growth, virescence, and phyllody. The majority of the symptomatic plants (10 out of 19) were infected with ‘*Ca*. P. solani’ in a mixed infection with ‘*Ca*. P. fraxini’ and ‘*Ca*. P. ziziphi’; it was also present in a single infection in three of the ten symptomatic plants [31].

## 11. ‘*Ca*. P. hispanicum’ and ‘*Ca*. P. meliae’

The 16SrXIII ribosomal group is only reported on the American continent [138]; both ‘*Ca*. P. hispanicum’ and ‘*Ca*. P. meliae’ were identified with several genetic variations that seem to be related to the strain’s geographic distribution more than to crop strain differentiation as it is shown for many of the phytoplasma-associated diseases in this area of the world.

### 11.1. Argentina

A strain of ‘*Ca.* P. hispanicum’ (ribosomal subgroup XIII-F) was found in strawberry plants showing virescence and phyllody. Another strain of ‘*Ca.* P. hispanicum’ (16SrXIII-C phytoplasma) was detected in China tree plants with yellows symptoms [101,138]. ‘*Ca.* P. meliae’ (ribosomal subgroup 16SrXIII-G) was reported in Argentina infecting China tree (*M. azedarach*) and plum plants. The symptoms observed in both plant species were yellowing, reduced leaf size, and witches’ broom [52,139].

### 11.2. Bolivia

In China tree plants with yellows symptoms, phytoplasma 16SrXIII-C strain CbY1 was detected [58].

### 11.3. Brazil

Since 2006, the likelihood of the presence of phytoplasmas affiliated with the 16SXIII group in Brazil has been considered. The partial sequence of a phytoplasma closely related to group 16SrXIII was obtained from coconut trees with coconut dry bud rot disease, from samples collected in the Rio de Janeiro state. The association between the phytoplasma and the disease has been reported but the complete identification of the pathogen has not been achieved. In Brazil, phytoplasmas of group 16SrXIII have been thoroughly identified for the first time in association with papaya apical curl necrosis (PACN). The first symptoms can be seen usually 5 months after papaya seedlings are transplanted in the field and the disease incidence levels can be up to 75%. Melo et al. [140] have sequenced four strains, namely, PACN-Br1, PACN-Br2, PACN-Br3, and PACN-Br4 (GenBank acc. nos. JQ792171, EU719111, JX893518 and JX893519, respectively) which showed indistinguishable restriction patterns. The plant samples were collected from the fields in the Espírito Santo state, the major papaya-producing state in Brazil.

Representative species of the Brassicaceae family can host phytoplasmas of the 16SrXIII group. The stunt has been affecting cauliflower and broccoli plants in municipalities of the state of São Paulo. The disease is characterized by plant stunting, inflorescence malformation, leaf reddening, and phloem necrosis and was found in association with distinct phytoplasma groups. Eckstein et al. [20] reported broccoli stunt in association with 16SrXIII phytoplasmas. The sequences of three strains have been deposited in GenBank BSP-22, BSP-27, and BSP-33 (acc. nos. JX626328, JX626326 and JX626329, respectively). The strains BSP-27 and BSP-33 were affiliated with the subgroup H, in group 16SrXIII [21]. As for cauliflower stunt disease, virtual RFLP and phylogenetic analysis revealed the presence of a strain of subgroup 16SrXIII-E in symptomatic plants. From the same farm, located in the municipality of Sorocaba, in an attempt to identify a possible insect vector for the phytoplasma, the leafhopper *B. hebe* was the most abundant and it harbored 16SrXIII-E phytoplasmas, thus being regarded as a putative vector of the phytoplasma that infects cauliflower. Sequences of the cauliflower stunt phytoplasma strain (GenBank acc. no. JN818844) and the *B. hebe* phytoplasma strain (GenBank acc. no. JN818843) were deposited in GenBank [141]. *Thumbergia erecta* (Benth.) T. Anderson has been reported as a new host for phytoplasma 16SrXIII-E. The associated disease was observed in the state of Minas Gerais and was designated as *T. erecta* yellow leaf (TEYL) identified as ‘*Ca.* P. hispanicum’ [142].

**Figure 7:**
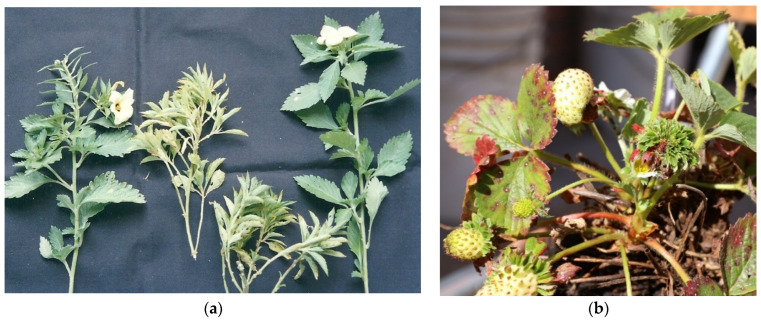
Phytoplasma symptoms in *T. ulmifolia* from Brazil in (**a**) and phyllody and witches’ broom symptoms in a strawberry plant from Chile in (**b**); both diseases are associated with 16SrXIII phytoplasmas.

The association of phytoplasmas and diseases in *Turnera ulmifolia* L. and in *Dimorphandra* spp. (*D. gardneriana* and *D. mollis*) has been known for more than a decade. Diseased *T. ulmifolia* plants were observed in the Rio de Janeiro state (southeast region) (Figure 7a), while diseased *Dimorphandra* spp. trees were from the state of Maranhão (Northeast region). Interestingly, both strains were identified as members of the 16SrXIII-A ribosomal group, on the basis of RFLP and sequencing analyses of the 16S rRNA gene. Yet, the analysis of the 16S rRNA genes has not enabled the phytoplasmas assignment to a ‘*Ca*. Phytoplasma’ species. Non-ribosomal genes (*leu*, *tufC*, *secA,* and *rp*) have been analyzed and suggested the possible assignment to ‘*Ca*. P. hispanicum’ or to a new phytoplasma taxon [143].

### 11.4. Chile

‘*Ca*. P. hispanicum’ (ribosomal subgroups 16SrXIII-F and 16SrXIII-K) was identified in Chilean strawberry plants as the 16SrXIII-F prevalent strain [144], which was also detected in calafate (*Berberis microphylla* G. Forst), mandarin, and sweet orange [145]. In strawberry plants, the symptoms observed were phyllody, achenes’ hypertrophy, and leaf reddening, independently of the presence of 16SrXIII-F or 16SrXIII-K strains. Generalized yellowing and untimely flowering symptoms appeared in one orange plant threadlike leaves and witches’ broom in one mandarin plant. The typical witches’ brooms have been associated with the presence of 16SrXIII-F strain in calafate plants. A draft genome sequence of 16SrXIII-F strain from Chilean strawberries was obtained and has been deposited in GenBank with acc. no. JAGVRH000000000 [146]. Epidemiological studies were carried out in strawberry fields, and the *Cixiosoma* sp., belonging to the Cixiidae family, a planthopper, was confirmed as a vector of 16SrXIII-F by transmission trials in periwinkle plants. Furthermore, one plant of *G. officinalis*, with chlorosis and shortened internodes, sampled in the vicinity of an infected strawberry field, was found positive for the same phytoplasma, suggesting that this species might serve as an alternative host species for the pathogen [147].

### 11.5. Paraguay

China tree is a tree species widely planted in Paraguay and in several countries in South America. The species is affected by phytoplasmas diseases as reported in Argentina, Bolivia, Brazil, and Paraguay. Diseased China trees exhibiting symptoms of decline, yellowing, and little leaf were observed in the cities of Caazapá, Villarrica, Itaguá, and Asunción. Phytoplasma identification was based on group-specific nested PCR and with RFLP with few restriction endonucleases. The results revealed the presence of phytoplasmas belonging to groups 16SrIII and 16SrXIII, in single and mixed infections. Single phytoplasma infection was verified in the samples from Caazapá (16SrXIII), Villarica (16SrXIII) and one sample from Itaguá (16SrIII). The phytoplasmas of either group were found in one sample collected in Itaguá and in the sample from Asunción. RFLP patterns identical to the subgroup 16SrIII-B were found for a phytoplasma from a tree sampled in Itaguá, while the RFLP pattern of the phytoplasma isolated from Caazapá resembled the subgroup 16SrXIII-C [101].

## 12. ‘*Ca*. P. brasiliense’

### 12.1. Brazil

**Figure 8:**
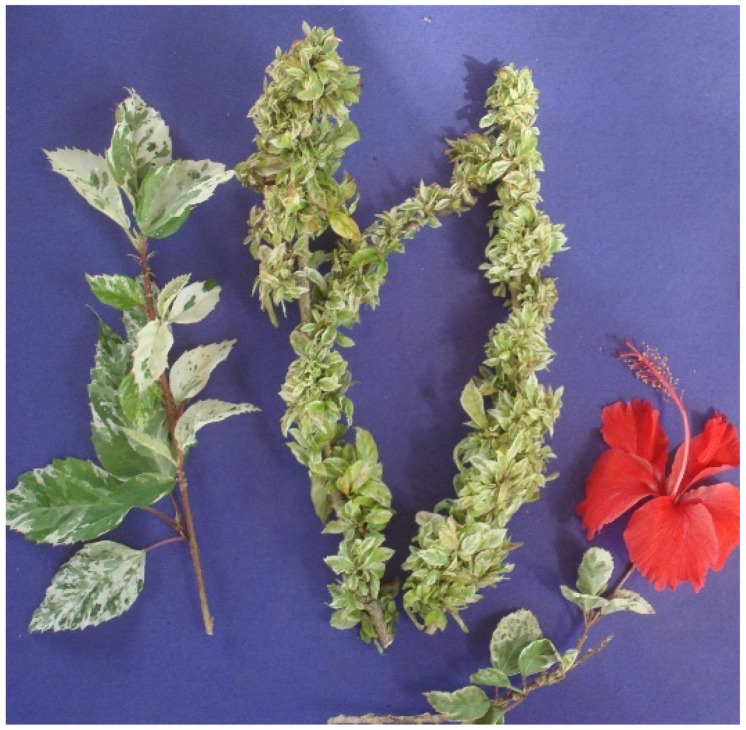
The two *Hibiscus* shoots in the center show symptoms of witches’ broom associated with the presence of ‘*Ca.* P. brasiliense’ (16SrXV-A) from Brazil.

This phytoplasma strain was detected in *H. rosa-sinensis* showing witches’ broom symptoms (strain HibWB26; GenBank acc. no. AF147708) (Figure 8) on the basis of unique properties of the 16S rRNA gene. Symptoms of the disease are characteristic of witches’ broom syndrome, such as leaf yellowing, short internodes, proliferation of shoots, and in some cases, premature flower dropping. The phytoplasma strain was from samples collected in the Rio de Janeiro state and is the representative of group 16SrXV, subgroup A [148]. Following the description of the 16SrXV phytoplasma group, naturally diseased plants of *C. roseus* exhibiting yellowing and witches’ broom symptoms were sampled in the state of Rio de Janeiro. Collective patterns obtained in RFLP analysis demonstrated that the strain in *C. roseus*, designated as HibWB-Cr, was indistinguishable from those of HibWB phytoplasma. In a chronologic order, *Sida* sp., cauliflower, and sunnhemp with symptoms of phytoplasma infection have been reported as hosts for the 16SrXV phytoplasmas. Being a weed and a phytoplasma host (GenBank acc. no. HQ230579), *Sida* sp. may be a source of inoculum to agronomic important plants since it grows in rows and fallow areas [149]. Cauliflower stunt has been reported in São Paulo state associated with phytoplasmas affiliated with the 16SrIII group. Conversely, stunted cauliflower plants from the state of Rio Grande do Sul became infected by a 16SrXV-A phytoplasma (GenBank acc. no. JN818845) [150]. In a broad survey to study the distribution of group 16SrIX phytoplasma in sunnhemp in São Paulo state, plants with shoot proliferation became infected by a 16SrXV phytoplasma strain (GenBank acc. no. KF878382) [28].

### 12.2. Peru

The first report of a GY disease was in a vineyard located in Piura, northwestern Peru, exhibiting symptoms of leaf yellowing, vein necrosis, and, in some cases, fruit shriveling. DNA samples of three plants with shriveled fruit tested phytoplasma-positive. The phytoplasma was designated as PeruGY, and was it was classified as a ‘*Ca.* P. brasiliense’. The strain sequences were deposited in GenBank under acc. nos. KX670807, KX670808 and KX670809 [151]. Papaya bunchy top (PBT) disease was observed on a papaya-producing farm in the same region as the grapevine. Symptoms on diseased plants included excessive proliferation of axillary shoots at the top or near the top of the main stem, shortening of internodes, leaf yellowing, and necrosis of leaf veins. The disease was associated with the presence of ‘*Ca.* P. brasiliense’. The strain sequences were designated as PeruPBT-1, PeruPBT-2 and PeruPBT-3 (GenBank acc. nos. KX810334, KX810335, KX810336, respectively) and the phytoplasma was classified as a variant of subgroup 16SrXV-B [152]. These PBT and GY phytoplasma strains may represent an emerging lineage distinct from the described ‘*Ca*. P. brasiliense’ strains in America.

## 13. Conclusions

There are about 50 ‘*Ca*. Phytoplasma’ species described worldwide, with 16 of them reported in South America; among the latter, three were reported only in this part of the world and seem to be endemic. The overall distribution of the 16 ‘*Ca*. Phytoplasma’ species associated with plant diseases in South America is summarized in Table 1. It appears that no reports are available from Venezuela, Guyana, and Suriname, very likely due to the lack of plant pathologists and/or the little impact of phytoplasma diseases on the agricultural situations of these countries. On the other hand, ‘*Ca*. P. lycopersici’, ‘*Ca.* P. sudamericanum’ and ‘*Ca*. P. meliae’ were only reported from the countries in South America where they seem to be endemic. However, ‘*Ca*. P. australasiae = australasiaticum’ and ‘*Ca*. P. tritici’ detected in a scattered manner are quite widespread on other continents, such as Asia and, partly, Europe. The verification of the world phytoplasma distribution is still far from being completed; therefore, considering the trading of agricultural propagation materials, it is possible they are present in other geographical areas.

In the above-listed countries, the ‘*Ca.* Phytoplasma’ species, most frequently detected in agricultural-relevant crops such as corn, alfalfa, grapevine, and other horticultural species, are ‘*Ca.* P. pruni’, ‘*Ca*. P. asteris’, and ‘*Ca.* P. fraxini’. However, there is still a strong need for clarifying the alternative host species and the insect vectors for several of these phytoplasmas to be able to evaluate the disease cycle and propose management strategies appropriately focused on reducing their spread in diverse agricultural situations.

It is clear that some ‘*Ca*. Phytoplasma’ species are more widespread than others and that they are usually found only on the American continent, suggesting that geographic distribution is the most important epidemiological criterium; however, the scattered and incidental detection of phytoplasmas mainly detected in other geographical areas of the world should not be overlooked, considering the potential of further spread, especially in case of their dissemination by widespread local, and still mainly unknown, insect vectors and, also, by both seeds and agamic propagation materials of agricultural plant species.

## Figures and Tables

**Table 1:** Summary of the ‘*Ca.* Phytoplasma’ species detected in South America.

‘*Ca*. Phytoplasma’ Species	Argentina	Bolivia	Brazil	Chile	Colombia	Ecuador	Paraguay	Peru	Uruguay
‘*Ca*. P. asteris’		X	X	X	X	X	X	X	
‘*Ca*. P. tritici’			X	X					
‘*Ca*. P. lycopersici’		X							
‘*Ca*. P. aurantifolia = citri’		X	X			X			
‘*Ca*. P. australasiae = australasiaticum		X						X	
‘*Ca.* P. pruni’	X	X	X	X	X		X	X	
‘*Ca.* P. ulmi’				X	X	X			
‘*Ca*. P. ziziphi’			X		X				
‘*Ca.* P. sudamericanum’			X						
‘*Ca.* P. fraxini’	X		X	X	X				
‘*Ca.* P. phoenicium’-related			X		X				
‘*Ca.* P. pyri’	X			X					X
‘*Ca.* P. solani’			X	X	X				
‘*Ca.* P. hispanicum’			X	X					
‘*Ca.* P. meliae’	X	X	X				X		
‘*Ca.* P. brasiliense’			X					X	

## Data Availability

Not applicable.

## References

[1] Bertaccini A., Duduk B., Paltrinieri S., Contaldo N. (2014). Phytoplasmas and phytoplasma diseases: A severe threat to agriculture. Am. J. Plant Sci..

[2] IRPCM (2004). ‘*Candidatus* Phytoplasma’, a taxon for the wall-less, non-helical prokaryotes that colonize plant phloem and insects. Int. J. Syst. Evol. Microbiol..

[3] Bertaccini A., Arocha-Rosete Y., Contaldo N., Duduk B., Fiore N., Montano H.G., Kube M., Kuo C.H., Martini M., Oshima K. (2022). Revision of the ‘*Candidatus* Phytoplasma’ species description guidelines. Int. J. Syst. Evol. Microbiol..

[4] Lee I.-M., Gundersen-Rindal D.E., Davis R.E., Bartoszyk I.M. (1998). Revised classification scheme of phytoplasmas based on RFLP analyses of 16S rRNA and ribosomal protein gene sequences. Int. J. Syst. Bacteriol..

[5] Bertaccini A. (2022). Plants and phytoplasmas: When bacteria modify plants. Plants.

[6] Jones P., Arocha Y., Antesana O., Montellano E., Franco P. (2005). “Brotes grandes” (big bud) of potato: A new disease associated with a 16SrI-B subgroup phytoplasma in Bolivia. Plant Pathol..

[7] Arocha Rosete Y., Antesana O., Montellano E., Franco P., Plata G., Jones P. (2007). ‘*Candidatus* Phytoplasma lycopersici’, a phytoplasma associated with “hoja de perejil” disease in Bolivia. Int. J. Syst. Evol. Microbiol..

[8] Kitajima E.W., Costa A.S. Microorganismos do tipo micoplasma associados ao enfezamento do milho. Proceedings of the 22a Reunião Anual da Sociedade Brasileira para o Progresso da Ciência, Universidade Federal da Bahia (UFBA).

[9] Bedendo I.P., Davis R.E., Dally E.L. (1997). Molecular evidence for the presence of maize bushy stunt phytoplasma in corn in Brazil. Plant Dis..

[10] Oliveira Sabato E., Oliveira C.M., Oliveira Sabato E. (2017). Corn stunting diseases. Diseases in Maize: Insect Vectors, Mollicutes and Viruses.

[11] Costa R.V., da Silva D.D., da Cota L.V., Campos L.J.M., Almeida R.E.M., de Bernardes F.P. (2019). Incidence of corn stunt disease in off-season corn hybrids in different sowing seasons. Pesqui. Agropecu. Bras..

[12] Oliveira C.M., Frizzas M.R. (2022). Eight decades of *Dalbulus maidis* (DeLong & Wolcott) (Hemiptera, Cicadellidae) in Brazil: What we know and what we need to know. Neotrop. Entomol..

[13] Silva E.G., Bedendo I.P., Casagrande M.V., Moraes V.A. (2009). Molecular identification and phylogenetic analysis of a group 16SrI-B phytoplasma associated with sugarcane yellow leaf syndrome in Brazil. J. Phytopathol..

[14] Boari A.J. (2008). Estudos Realizados Sobre o Amarelecimento Fatal do Dendezeiro (Elaeis guineensis Jacq).

[15] Bittencourt C.B., Lins P.C., Boari A.J., Quirino B.F., Teixeira W.G., Souza Junior M.T., Kamyab H. (2021). Oil palm fatal yellowing (FY), a disease with an elusive causal agent. Elaeis guineensis.

[16] Boari A.J., Costa A.S.V.C., Souza C.A. (2010). Variabilidade de fitoplasmas associados ao amarelecimento fatal do dendezeiro (*Elaeis guineensis* Jacq.) com base no gene 16S rDNA. Proceedings of the Congresso da Rede Brasileira de Tecnologia de Biodiesel 4, Congresso Brasileiro de Plantas Oleaginosas, Óleos, Gorduras e Biodiesel 7—Biodiesel: Inovação Tecnológica e Qualidade.

[17] Souza C.A., Boari A.J., Costa A.S.V., Fernandes P.F.L. (2010). Incidência de fitoplasma associado ao dendezeiro com amarelecimento fatal no estado do Pará. Proceedings of the Congresso da Rede Brasileira de Tecnologia de Biodiesel 4, Congresso Brasileiro de Plantas Oleaginosas, Óleos, Gorduras e Biodiesel 7—Biodiesel: Inovação Tecnológica e Qualidade.

[18] Pereira T.B.C., Bedendo I.P. (2017). A ‘*Candidatus* Phytoplasma asteris’ (16SrI group) phytoplasma associated with delayed maturity in soybean plants in Brazil. Australas. Plant Dis. Notes.

[19] Silva E.G., Flôres D., Bedendo I.P. (2015). Bougainvillea shoot proliferation, a new disease induced by distinct phytoplasmas. J. Phytopathol..

[20] Eckstein B., Barbosa J.C., Kreyci P.F., Canale M.C., Brunelli K.R., Bedendo I.P. (2013). Broccoli stunt, a new disease in broccoli plants associated with three distinct phytoplasma groups in Brazil. J. Phytopathol..

[21] Pérez-López E., Luna-Rodríguez M., Olivier C.Y., Dumonceaux T.J. (2016). The underestimated diversity of phytoplasmas in Latin America. Int. J. Syst. Evol. Microbiol..

[22] Canale M.C., Mello A.P.O.A., Martins T., Bedendo I.P. (2012). Patossistema fitoplasma-brássicas: Identificação do agente em repolho, couve-flor e brócolis, potenciais vetores e epidemiologia. Rev. Anu. Patol. Plant.

[23] Bianchini L., Bedendo I.P. (2000). Presença de um fitoplasma do grupo I, evidenciado por PCR, em plantas de erigeron (*Erigeron bonariensis* L.) com superbrotamento. Fitopatol. Bras..

[24] Neroni R.C., Bedendo I.P., Kuniyuki H. (2006). Identificação molecular de fitoplasmas associados ao amarelo da videira. Fitopatol. Bras..

[25] Melo L.A., Ventura J.A., Costa H., Kitajima E.W., Bedendo I.P. (2007). Identificação molecular de fitoplasmas associados a filodia do morangueiro no Brasil. Fitopatol. Bras..

[26] Alves M.S., Ribeiro G.M., Souza A.N., Carvalho C.M. (2017). First report of a phytoplasma associated with witches’ broom symptoms in *Waltheria indica* in Brazil. New Dis. Rep..

[27] Alves M.S., Ribeiro G.M., Souza A.N., Carvalho C.M. (2018). First report of a ‘*Candidatus* Phytoplasma asteris’ isolate associated with *Macroptilium lathyroides* yellow leaf disease in Brazil. New Dis. Rep..

[28] Bianco L.F., Martins E.C., Toloy R.S., Coletti D.A.B., Teixeira D.C., Wulff N.A. (2014). Fisrt report of phytoplasmas groups 16SrI and 16SrXV in *Crotalaria juncea* in Brazil. Plant Dis..

[29] Gajardo A., Fiore N., Prodan S., Paltrinieri S., Botti S., Pino A.M., Zamorano A., Montealegre J., Bertaccini A. (2009). Phytoplasmas associated with grapevine yellows disease in Chile. Plant Dis..

[30] Alvarez E., Mejía J.F., Contaldo N., Paltrinieri S., Duduk B., Bertaccini A. (2014). ‘*Candidatus* Phytoplasma asteris’ strains associated with oil palm lethal wilt in Colombia. Plant Dis..

[31] Franco-Lara L., Contaldo N., Mejía J.F., Paltrinieri S., Duduk B., Bertaccini A. (2017). Detection and identification of phytoplasmas associated with declining *Liquidambar styraciflua* trees in Colombia. Trop. Plant Pathol..

[32] Perilla Henao L.M., Dickinson M., Franco-Lara L. (2012). First report of ‘*Candidatus* Phytoplasma asteris’ affecting woody hosts (*Fraxinus uhdei*, *Populus nigra*, *Pittosporum undulatum* and *Croton* spp.) in Colombia. Plant Dis..

[33] Castillo Carrillo C., Paltrinieri S., Buitrón Bustamante I., Bertaccini A. (2018). Detection and molecular characterization of a 16SrI-F phytoplasma in potato showing purple top disease in Ecuador. Australas. Plant Pathol..

[34] Ganem Junior E.J., Segnana L.G., Kitajima E.W., Bedendo I.P. (2019). Sesame phyllody associated with a 16SrI-B phytoplasma, a ‘*Candidatus* Phytoplasma asteris’-related strain, in Paraguay. Sci. Agric..

[35] Lezcano R., Machado V. (1997). Fitoplasmas y espiroplasmas de maiz em el Paraguay. Fitopatol. Bras..

[36] Alvarez E., Pardo J.M., Contaldo N., Paltrinieri S., Bertaccini A., Mejia J.F. Phytoplasma infections in cassava plants with frog skin disease from Costa Rica and Paraguay. Proceedings of the IOM 2014.

[37] Hodgetts J., Chuquillangui C., Muller G., Arocha Y., Gamarra D., Pinillos O., Velit E., Lozada P., Boa E., Boonham N. (2009). Surveys reveal the occurrence of phytoplasmas in plants at different geographical locations in Peru. Ann. Appl. Biol..

[38] Gamarra D.G., Milagros Villar C., Torres Suarez G., Ingaruca Esteban W.D., Contaldo N., Carrasco Lozano E.C., Bertaccini A. (2022). Diverse phytoplasmas associated with maize bushy stunt disease in Peru. Eur. J. Plant Pathol..

[39] Rodrigues Jardim B., Tran-Nguyen L.T.T., Gambley C., Al-Sadi A.M., Al-Subhi A.M., Foissac X., Salar P., Cai H., Yang J.-Y., Davis R. (2023). The observation of taxonomic boundaries for the 16SrII and 16SrXXV phytoplasmas using genome-based delimitation. Int. J. Syst. Evol. Microbiol..

[40] Jones P., Arocha Y., Antesana O., Montillano E., Franco P. (2005). First report of an isolate of ‘*Candidatus* Phytoplasma australiense’ associated with a yellow leaf roll disease of peach (*Prunus persicae*) in Bolivia. Plant Pathol..

[41] Arocha Y., Plata G., Franco J., Maín G., Veramendi S., Lazcano F., Crespo J.L., Lino V., Calderón C., Llerena R. (2010). Occurrence of ‘*Candidatus* Phytoplasma aurantifolia’ (16SrII group) in Bolivia. Plant Pathol..

[42] Barros T.S.L., Kitajima E.W., Resende R.O. (1998). Diversidade de isolados brasileiros de fitoplasmas através da análise do 16S rDNA. Fitopatol. Bras..

[43] Bedendo I.P., Davis R.E., Dally E.L. (1999). Detecção e caracterização de fitoplasmas em plantas de vinca (*Catharanthus roseus*) e de pimenta (*Capsicum frutescens*) através das técnicas de duplo PCR e de RFLP. Summa Phytopathol..

[44] Mafia R.G., Barreto R.W., Vanetti C.A., Hodgetts J., Dickinson M., Alfenas A.C. (2008). A phytoplasma associated with witches’ broom disease of *Tabebuia pentaphylla* in Brazil. Plant Pathol..

[45] Souza A.N., Carvalho S.L., Silva F.N., Alfenas A.C., Zauza E.A.V., Carvalho C.M. (2015). First report of phytoplasma associated with *Eucalyptus urophylla* showing witches’ broom in Brazil. Phytopathog. Mollicutes.

[46] Silva F.N., Queiroz R.B., Souza A.N., Al-Sadi A.M., Siqueira D.L., Elliot S.L., Carvalho C.M. (2014). First report of a 16SrII-C phytoplasma associated with asymptomatic acid lime (*Citrus aurantifolia*) in Brazil. Plant Dis..

[47] Donkersley P., Silva F.W.S., Alves M.S., Carvalho C.M., Al-Sadi A.M., Elliot S.L., Topolovec-Pintarić S. (2019). Asymptomatic phytoplasma reveal a novel and troublesome infection. Plant Diseases—Current Threats and Management Trends.

[48] Caicedo J., Crized M., Pozo A., Cevallos A., Simbalo L., Rivera L., Arahana V. (2015). First report of ‘*Candidatus* Phytoplasma aurantifolia’ (16SrII) associated with potato purple top in San Gabriel-Carchi, Ecuador. New Dis. Rep..

[49] Giaccaglia G., Castillo-Carrillo C., Pacini F., Bertaccini A. (2024). Phloem limited bacteria in potato with purple top disease and in *Bactericera cockerelli* in Ecuador. Phytopathog. Mollicutes.

[50] Galdeano E., Torres L.E., Meneguzzi N., Guzmán F., Gomez G.G., Docampo D.M., Conci L.R. (2004). Molecular characterization of 16S ribosomal DNA and phylogenetic analysis of two X-disease group phytoplasmas affecting China-tree (*Melia azedarach* L.) and garlic (*Allium sativum* L.) in Argentina. J. Phytopathol..

[51] Curzel V., Fernández F., Conci L., Bejarano N. (2021). Advances in the research on peach yellows disease, in the productive areas of Jujuy, Argentina. Agrocienc. Urug..

[52] Bongiorno V., Alessio F., Curzel V., Nome C., Fernández F.D., Conci L.R. (2020). ‘*Ca*. Phytoplasma pruni’ and ‘*Ca*. Phytoplasma meliae’ are affecting plum in Argentina. Australas. Plant Dis. Notes.

[53] Galdeano E., Guzmán F.A., Fernández F., Conci R.G. (2013). Genetic diversity of 16SrIII group phytoplasmas in Argentina. Predominance of subgroups 16SrIII-J and B and two new subgroups 16SrIII-W and X. Eur. J. Plant Pathol..

[54] Guzmán F., Giolitti F., Fernández F., Nome C., Lenardon S., Conci L. (2014). Identification and molecular characterization of a phytoplasma associated with sunflower in Argentina. Eur. J. Plant Pathol..

[55] Fernández F.D., Uset A., Baumgratz G., Conci L.R. (2018). Detection and identification of a 16SrIII-J phytoplasma affecting cassava (*Manihot esculenta* Crantz) in Argentina. Australas. Plant Dis. Notes.

[56] Férnandez F.D., Guzmán F.A., Baffoni P., Reinoso L., Kiehr M., Delhey R., Favere V.M., Galdeano E., Conci L.R. (2020). Phytoplasmas of subgroup 16SrIII-J associated with *Beta vulgaris* in Argentina. Trop. Plant Pathol..

[57] Fernandez F.D., Carloni E., Alessio F., Bongiorno V., Conci L.R. (2022). First report of a 16SrIII-X phytoplasma associated with *Lactuca sativa* witches’ broom in Argentina. New Dis. Rep..

[58] Harrison N.A., Boa E., Carpio M.L. (2003). Characterization of phytoplasmas detected in Chinaberry trees with symptoms of leaf yellowing and decline in Bolivia. Plant Pathol..

[59] Arocha Rosete Y., Plata G., Franco J., Maín G., Veramendi S., Lazcano F., Crespo J.L., Lino V., Calderón C., Llerena R. (2010). First report of a 16SrIII phytoplasma (X-disease group) affecting bell pepper, strawberry (frutilla), *Schinus molle* and *Arracacia xanthorrhiza* in Cochabamba, Bolivia. Plant Pathol..

[60] Flôres D., Haas I.C., Canale M.C., Bedendo I.P. (2013). Molecular identification of a 16SrIII-B phytoplasma associated with cassava witches’ broom disease. Eur. J. Plant Pathol..

[61] Flôres D., Bedendo I.P. (2013). A subgroup 16SrIII-B phytoplasma identified in honeyweed plants with leaf deformation in Brazil. Australas. Plant Dis. Notes.

[62] Oliveira S.A.S., Abreu E.F.M., Araújo T.S.E., Oliveira J., Andrade E.C., Garcia J.M.P., Álvarez E. (2014). First report of a 16SrIII-L phytoplasma associated with frogskin disease in cassava (*Manihot esculenta* Crantz) in Brazil. Plant Dis..

[63] Souza A.N., Silva F.N., Bedendo I.P., Carvalho C.M. (2014). A phytoplasma belonging to a 16SrIII-A subgroup and dsDNA virus associated with cassava frogskin disease in Brazil. Plant Dis..

[64] Amaral Mello A.P.O., Eckstein B., Flôres D., Kreyci P.F., Bedendo I.P. (2011). Identification by computer-simulated RFLP of phytoplasmas associated with eggplant giant calyx representative of two subgroups, a lineage of 16SrIII-J and the new subgroup 16SrIII-U. Int. J. Syst. Evol. Microbiol..

[65] Amaral Mello A.P.O., Bedendo I.P., Kitajima E.W., Ribeiro L.F., Kobori R. (2006). Tomato big bud associated with a phytoplasma belonging to group 16SrIII in Brazil. Int. J. Pest Manag..

[66] Teixeira D.d.C., Wulff N.A., Martins E.C., Kitajima E.W., Bassanezi R., Ayres A.J., Eveillard S., Saillard C., Bové J.-M. (2008). A phytoplasma closely related to the pigeon pea witches’-broom phytoplasma (16Sr-IX) is associated with citrus “huanglongbing” symptoms in the state of São Paulo, Brazil. Phytopathology.

[67] Wulff N.A., Fassini C.G., Marques V.V., Martins E.C., Coletti D.A.B., Teixeira D.d.C., Sanches M.M., Bové J.-M. (2019). Molecular characterization and detection of 16SrIII group phytoplasma associated with “huanglongbing” symptoms. Phytopathology.

[68] Barbosa J.C., Gasparoto M.C.G., Eckstein B., Bergamin Filho A., Bedendo I.P. (2021). Potential reservoirs of a ‘*Candidatus* Phytoplasma pruni’-related strains (16SrIII-X) associated with HLB-like symptoms in citrus in Brazil. Trop. Plant Pathol..

[69] Fugita J.M.S., Pereira T.B.C., Banzato T.C., Kitajima E.W., Souto E.R., Bedendo I.P. (2017). Occurrence of a subgroup 16SrIII-J phytoplasma in non-symptomatic *Brachiaria decumbens* cultivated in a grazing area. Trop. Plant Pathol..

[70] Banzato T.C., Ferreira J., Bedendo I.P. (2021). Field mustard (*Brassica rapa*) an invasive weed species in cauliflower fields is a host of multiple phytoplasmas. Australas. Plant Pathol..

[71] Kitajima E.W., Robbs C.F., Kimura O.O. (1981). Irizado do chuchuzeiro e o superbrotamento do maracujá: Duas enfermidades associadas a microrganismos do tipo micoplasma constatadas nos estados do Rio de Janeiro e Pernambuco. Fitopatol. Bras..

[72] Davis R.E., Zhao Y., Dally E.L., Jomantiene R., Lee I.-M., Wei W., Kitajima E.W. (2012). ‘*Candidatus* Phytoplasma sudamericanum’, a novel taxon, and strain PassWB-Br4, a new subgroup 16SrIII-V phytoplasma, from diseased passion fruit (*Passiflora edulis* f. *flavicarpa* Deg.). Int. J. Syst. Evol. Microbiol..

[73] Ferreira J., Almeida C.A., Pereira T.B.C., Favara G., Bedendo I.P. (2022). Acerola shoot proliferation induced by a phytoplasma enclosed in the subgroup 16SrIII-F. Sci. Agric..

[74] Oliveira F.F., Ferreira J., Galvão S.R., Kitajima E.W., Bedendo I.P. (2020). Chrysanthemum is a new host of a group 16SrIII phytoplasma (16SrIII-X) that induces colour-breaking in affected plants. Eur. J. Plant Pathol..

[75] Montano H.G., Davis R.E., Dally E.L., Pimentel J.P., Brioso P.S.T. (2000). Identification and phylogenetic analysis of a new phytoplasma from diseased chayote in Brazil. Plant Dis..

[76] Alves M.S., Souza A.N., Ribeiro G.M., Carvalho C.M. (2017). First report of a 16SrIII-B phytoplasma associated with *Momordica charantia* witches’ broom in Brazil. Plant Dis..

[77] Montano H.G., Brioso P.S.T., Cunha Júnior J.O., Figueiredo D.V., Pimentel J.P. (2007). First report of group 16SrIII phytoplasma in loofah (*Luffa cylindrica*). Bull. Insectol..

[78] Montano H.G., Brioso P.S.T., Pimentel J.P., Figueiredo D.V., Cunha Júnior J.O. (2006). *Cucurbita moschata*, new phytoplasma host in Brazil. J. Plant Pathol..

[79] Montano H.G., Brioso P.S.T., Pereira R.C., Pimentel J.P. (2007). *Sicana odorifera*, a new phytoplasma host. Bull. Insectol..

[80] Montano H.G., Bertaccini A., Pimentel J.P., Mejia J.F., Contaldo N., Paltrinieri S. (2015). *Lagenaria siceraria* yellows associated with phytoplasma in Brazil. Phytopathog. Mollicutes.

[81] Montano H.G., Arocha Rosete Y. (2019). First report of the identification of a ‘*Candidatus* Phytoplasma pruni’-related strain of phytoplasma in *Melothria pendula*. New Dis. Rep..

[82] Rapussi M.C.C., Eckstein B., Flôres D., Haas I.C.R., Amorim L., Bedendo I.P. (2012). Cauliflower stunt associated with a phytoplasma of subgroup 16SrIII-J and the spatial pattern of the disease. Eur. J. Plant Pathol..

[83] Kreyci P.F., Eckstein B., Lopes J.R.S., Ferreira J., Bedendo I.P. (2018). Transmission of ‘*Candidatus* Phytoplasma pruni’-related strain associated with broccoli stunt by four species of leafhoppers. J. Phytopathol..

[84] Eckstein B., Silva E.G., Bedendo I.P. (2012). Shoot proliferation and leaf malformation of *Celosia argentea* and *Celosia spicata* caused by a phytoplasma of the 16SrIII-J group. J. Phytopathol..

[85] Munhoz E.M., Pereira T.B.C., Silva E.M.S., Bedendo I.P. (2016). A subgroup 16SrIII-J phytoplasma associated with A*egiphila verticillata*, a typical wild plant of Brazilian savanna region, exhibiting witches’ broom symptoms. Trop. Plant Pathol..

[86] Duarte V., Silva E.G., Hass I.C.R., Bedendo I.P., Kitajima E.W. (2009). First report of a group 16SrIII-B phytoplasma associated with decline of China tree in Brazil. Plant Dis..

[87] Ribeiro L.F., Mello A.P.A., Bedendo I.P., Gioria R. (2006). Phytoplasma associated with shoot proliferation in begonia. Sci. Agric..

[88] Ribeiro L.F., Bedendo I.P. (2006). Proliferação de ramos em plantas comerciais de bico-de-papagaio associada a fitoplasma do grupo 16SrIII. Fitopatol. Bras..

[89] Ribeiro L.F., Bedendo I.P., Sanhueza R.M.V. (2007). Evidência molecular da ocorrência de um fitoplasma associado ao lenho mole da macieira. Summa Phytopathol..

[90] Melo L.A., Bedendo I.P., Yuki V. (2009). Abobrinha-de-moita: Um novo hospedeiro de fitoplasma do grupo 16SrIII. Trop. Plant Pathol..

[91] Fiore N., González X., Zamorano A., Quiroga N., Paillalef R., Pino A.M. (2015). Phytoplasmas associated with yellow wilt disease of sugar beet in Chile. Phytopathog. Mollicutes.

[92] Fiore N., Zamorano A., Pino A.M. (2015). Identification of phytoplasmas belonging to the ribosomal groups 16SrIII and 16SrV in Chilean grapevines. Phytopathog. Mollicutes.

[93] Quiroga N., Bustamante M., Gamboa C., Molina J., Zamorano A., Fiore N. (2017). 16SrIII-J phytoplasmas infecting lettuce and Swiss chard crops in Chile. Phytopathog. Mollicutes.

[94] Quiroga N., Longone V., González X., Zamorano A., Pino A.M., Picciau L., Alma A., Paltrinieri S., Contaldo N., Bertaccini A. (2019). Transmission of 16SrIII-J phytoplasmas by the leafhoppers *Paratanus exitiousus* and *Bergallia valdiviana*. Phytopathol. Mediterr..

[95] Quiroga N., Gamboa C., Soto D., Pino A.M., Zamorano A., Campodonico J., Alma A., Bertaccini A., Fiore N. (2020). Update and new epidemiological aspects about grapevine yellows in Chile. Pathogens.

[96] Zamorano A., Fiore N. (2016). Draft genome sequence of 16SrIII-J phytoplasma, a plant pathogenic bacterium with a broad spectrum of hosts. Genome Announc..

[97] Alvarez E., Mejía J.F., Llano G., Loke J., Calari A., Duduk B., Bertaccini A. (2009). Characterization of a phytoplasma associated with frogskin disease in cassava. Plant Dis..

[98] Calari A., Paltrinieri S., Duduk B., Mejia J.F., Alvarez E., Carraro L., Bertaccini A. Genomic variability within conserved genes of 16SrIII phytoplasma strains after long term in vitro maintenance in periwinkle. Proceedings of the IOM 17th International Congress.

[99] Galvis C.A., Leguizamón J.E., Gaitán Á.L., Mejía J.F., Álvarez E., Arroyave J. (2007). Detection and identification of a 16SrIII-related phytoplasma associated with coffee crispiness disease in Colombia. Plant Dis..

[100] Cardozo Téllez L., Pardo J.M., Zacher M., Torres A., Álvarez E. (2016). First report of a 16SrIII phytoplasma associated with frogskin disease in cassava (*Manihot esculenta*), in Paraguay. Plant Dis..

[101] Arneodo J.D., Galdeano E., Orrego A., Stauffer A., Nome S.F., Conci L.R. (2005). Identification of two phytoplasmas detected in China-trees with decline symptoms in Paraguay. Australas. Plant Pathol..

[102] Torres Suarez G., Gamarra D.G., Villar C.M., Llacza Munive S.L., Satta E., Carrasco Lozano E.C., Bertaccini A. (2021). Detection and identification of a 16SrIII-J subgroup phytoplasma associated with faba bean in Peru. J. Phytopathol..

[103] Amaral Mello A.P.O., Ribeiro L.F.C., Massola Júnior N.S., Bedendo I.P. (2004). Um fitoplasma do grupo 16SrV associado ao superbrotamento da crotalaria. Summa Phytopathol..

[104] Arismendi N.L., Riegel R., Carrillo R. (2014). *In vivo* transmission of ‘*Candidatus* Phytoplasma ulmi’ by *Amplicephalus curtulus* (Hemiptera: Cicadellidae) and its effect on ryegrass (*Lolium multiflorum* Cv. Tama). J. Econ. Entomol..

[105] Quiroga N., Gamboa C., Medina G., Contaldo N., Torres F., Bertaccini A., Zamorano A., Fiore N. (2022). Survey for ‘*Candidatus* Liberibacter’ and ‘*Candidatus* Phytoplasma’ in citrus in Chile. Pathogens.

[106] Mejia J.F., Contaldo N., Paltrinieri S., Pardo J.M., Rios C.A., Alvarez E., Bertaccini A. (2011). Molecular detection and identification of group 16SrV and 16SrXII phytoplasmas associated with potatoes in Colombia. Bull. Insectol..

[107] Ribeiro L.F., Silva E.G., Bedendo I.P. (2008). Evidência molecular da ocorrência de fitoplasma associado ao superbrotamento do maracujazeiro em cinco estados brasileiros. Trop. Plant Pathol..

[108] Fernández F.D., Conci V.C., Kirschbaum D.S., Conci L.R. (2013). Molecular characterization of a phytoplasma of the ash yellows group occurring in strawberry (*Fragaria x ananassa* Duch.) plants in Argentina. Eur. J. Plant Pathol..

[109] Conci L., Meneguzzi N., Galdeano E., Torres L., Nome C., Nome S. (2005). Detection and molecular characterization of an alfalfa phytoplasma in Argentina that represents a new subgroup in the 16S rDNA ash yellows group (‘*Candidatus* Phytoplasma fraxini’). Eur. J. Plant Pathol..

[110] Flôres D., Amaral Mello A.P.O., Pereira T.B.C., Rezende J.A.M., Bedendo I.P. (2015). A novel subgroup 16SrVII-D phytoplasma identified in association with erigeron witches’ broom. Int. J. Syst. Evol. Microbiol..

[111] Meneguzzi N.G., Torres L.E., Galdeano E., Guzmán F.A., Nome S.F., Conci L.R. (2008). Molecular characterization of a phytoplasma of the ash yellows group (16SrVII-B) occurring in *Artemisia annua* and *Conyza bonariensis* weeds. Agriscientia.

[112] Barros T.S.L., Davis R.E., Resende R.O., Dally E.L. (2002). Erigeron witches’-broom phytoplasma in Brazil represents new subgroup VII-B in 16S rRNA gene group VII, the ash yellows phytoplasma group. Plant Dis..

[113] Pereira T.B.C., Dally E.L., Davis R.E., Banzato T.C., Bedendo I.P. (2016). Ming aralia (*Polyscias fruticosa*), a new host of a phytoplasma subgroup 16SrVII-B strain in Brazil. Plant Dis..

[114] Pereira T.B.C., Dally E.L., Davis R.E., Banzato T.C., Galvão S.R., Bedendo I.P. (2016). Cauliflower is a new host of a subgroup 16SrVII-B phytoplasma associated with stunting disease in Brazil. Plant Dis..

[115] Ferreira J., Almeida C.A., Oliveira F.F., Fariña A., Kitajima E.W., Bedendo I.P. (2022). Phytoplasma of 16SrVII-B subgroup associated to shoot proliferation in *Physalis peruviana* plants. Sci. Agric..

[116] Ferreira J., Pereira T.B.C., Almeida C.A., Bedendo I.P. (2021). Olive tree represents a new host of a subgroup 16SrVII-B phytoplasma associated with witches’ broom disease in Brazil. Plant Dis..

[117] Flôres D., Amaral Mello A.P.O., Massola Júnior N.S., Bedendo I.P. (2013). First report of a group 16SrVII-C phytoplasma associated with shoot proliferation of sunnhemp (*Crotalaria juncea*) in Brazil. Plant Dis..

[118] Fugita J.M.S., Pereira T.B.C., Banzato T.C., Kitajima E.W., Souto E.R., Bedendo I.P. (2017). Molecular characterization of a phytoplasma affiliated with the 16SrVII group representative of the novel 16SrVII-F subgroup. Int. J. Syst. Evol. Microbiol..

[119] Barbosa J.C., Eckstein B., Inoue Nagata A.K., Bergamin Filho A., Bedendo I.P. (2022). Molecular delineation of a phytoplasma representative of the novel 16SrVII G subgroup found in citrus trees with huanglongbing symptoms. J. Plant Dis. Prot..

[120] Arismendi N., González F., Zamorano A., Andrade N., Pino A.M., Fiore N. (2011). Molecular identification of ‘*Candidatus* Phytoplasma fraxini’ in murta and peony in Chile. Bull. Insectol..

[121] Filgueira J.J., Franco_lara L., Salcedo J.E., Gaitan S.L., Boa E.R. (2004). Urapan (Fraxinus udhei) dieback, a new disease associated with a phytoplasma in Colombia. Plant Pathol..

[122] Wulff N.A., Teixeira D.C., Martins E.C., Toloy R.S., Bianco L.F., Colletti D.A.B., Kitajima E.W., Bové J.-M. (2015). Sunnhemp, a major source-plant of the phytoplasma associated with “huanglongbing” symptoms of sweet orange in São Paulo state, Brazil. J. Citrus Pathol..

[123] Sanches M.M., Wulff N.A., Ferreira E.A., Santos J.F., Angarten M.B.O., Carbonari J.J., Oliveira R.P., Ishida A.K.N., Martins O.M. (2016). Survey for phytoplasmas and ‘*Candidatus* Liberibacter sp.’ from HLB-like symptomatic citrus plants in Brazil. Citrus Res. Technol..

[124] Marques R.N., Teixeira D.C., Yamamoto P.T., Lopes J.R.S. (2012). Weedy hosts and prevalence of potential leafhopper vectors (Hemiptera: Cicadellidae) of a phytoplasma (16SrIX group) associated with “huanglongbing” symptoms in citrus groves. J. Econ. Entomol..

[125] Lopes-da-Silva M., Martins O.M., Wulff N.A., Ichida C.M., Guimarães A.S., Guimarães G.C., de Souza S.L.B., Sanches M.M. (2020). Survey of 16SrIX phytoplasmas associated with HLB-symptoms in weeds and leafhoppers at citrus orchards. Trop. Plant Pathol..

[126] Barbosa J.C., Eckstein B., Bergamin Filho A., Bedendo I.P., Kitajima E.W. (2012). Molecular characterization of a phytoplasma of group 16SrIX related to ‘*Ca*. Phytoplasma phoenicium’ in periwinkle in Brazil. Trop. Plant Pathol..

[127] Duduk B., Mejia J.F., Calari A., Bertaccini A. Identification of 16SrIX group phytoplasmas infecting Colombian periwinkles and molecular characterization on several genes. Proceedings of the IOM 17th International Congress.

[128] Fernández F.D., Marini D., Farrando R., Conci L.R. (2017). First report of a ‘*Candidatus* Phytoplasma pyri’ strain in Argentina. Australas. Plant Dis. Notes.

[129] Fernández F.D., Conci V.C. (2019). First report of ‘*Candidatus* Phytoplasma pyri’-related strain causing pear decline in Argentina. Crops Prot..

[130] Facundo R., Quiroga N., Méndez P., Zamorano A., Fiore N. (2017). First report of ‘*Candidatus* Phytoplasma pyri’ in pear in Chile. Plant Dis..

[131] Maeso D.C., Martínez A., Federici M.T., Gonçalves L., Silvera M., Cabrera D., Núñez S., Walasek W., Giunchedi L. (2012). El decaimiento del peral en Uruguay: Generalidades y trabajos experimentales de INIA Las Brujas. INIA Las Brujas—Estación Experimental “Wilson Ferreira Aldunate”.

[132] Maeso D.C. (2015). El decaimiento del peral. Aportes experimentales. Rev. INIA.

[133] Valle D., Burckhardt D., Mujica V., Zoppolo R., Morelli E. (2017). The occurrence of the pear psyllid, *Cacopsylla bidens* (Šulc, 1907) (Insecta: Hemiptera: Psyllidae), in Uruguay. Check List.

[134] Maeso Tozzi D.C. (2020). El Decaimiento del Peral en Uruguay: Aspectos Generales y Trabajos Experimentales de INIA Las Brujas.

[135] Maeso D.C. (2014). El decaimiento del peral: Un problema adicional en ciertas combinaciones variedad-portainjerto. Rev. INIA.

[136] Montano H.G., Contaldo N., David T.V.A., Silva I.B., Paltrinieri S., Bertaccini A. (2011). Hibiscus witches’ broom disease associated with different phytoplasma taxa in Brazil. Bull. Insectol..

[137] Paltrinieri S., Botti S., Dal Molin F., Mori N., Fiore N., Bertaccini A. (2006). Are phytoplasmas involved in a severe peach decline?. Acta Hortic..

[138] Fernández F.D., Meneguzzi N.G., Guzmán F.A., Kirschbaum D.S., Conci V.C., Nome C.F., Conci L.R. (2015). Detection and identification of a novel 16SrXIII subgroup phytoplasma associated with strawberry red leaf disease in Argentina. Int. J. Syst. Evol. Microbiol..

[139] Fernández F.D., Galdeano E., Kornowski M.V., Arneodo J.D., Conci L.R. (2016). Description of ‘*Candidatus* Phytoplasma meliae’, a phytoplasma associated with Chinaberry (*Melia azedarach* L.) yellowing in South America. Int. J. Syst. Evol. Microbiol..

[140] Melo L., Silva E., Flôres D., Ventura J., Costa H., Bedendo I. (2013). A phytoplasma representative of a new subgroup, 16SrXIII-E, associated with papaya apical curl necrosis. Eur. J. Plant Pathol..

[141] Canale M.C., Bedendo I.P. (2020). Report of ‘*Candidatus* Phytoplasma hispanicum’ (16SrXIII-E) associated with cauliflower stunt in São Paulo state, Brazil, and *Balclutha hebe* as its potential vector. Plant Dis..

[142] Alves M.S., Souza A.N., Ribeiro G.M., Xavier A.S., Carvalho C.M. (2016). A 16SrXIII-E subgroup phytoplasma is associated with *Thunbergia erecta* yellow leaf in Brazil. Australas. Plant Dis. Notes.

[143] Contaldo N., Pacini F., Montano H.G., Pimentel J.P., Bertaccini A. (2023). Multigene analyses for identification of phytoplasma strains infecting *Dimorphandra gardneriana* and *Turnera ulmifolia* in Brazil. Phytopathog. Mollicutes.

[144] Cui W., Quiroga N., Curkovic S.T., Zamorano A., Fiore N. (2019). Detection and identification of 16SrXIII-F and a novel 16SrXIII phytoplasma subgroups associated with strawberry phyllody in Chile. Eur. J. Plant Pathol..

[145] Madariaga M., Ramírez I. (2019). Identification of a phytoplasma associated with witches’ broom symptoms in calafate (*Berberis microphylla* G. Forst.). Chil. J. Agric. Res..

[146] Cui W., Zamorano A., Fiore N. (2022). Draft genome sequence resource of ‘*Fragaria × ananassa*’ phyllody phytoplasma strain StrPh-CL from Chilean strawberry. Plant Dis..

[147] Cui W., Muñoz V., Navarrete M., Cabrera S., Campodonico J., Estrada M., Zamorano A., Fiore N. (2024). Insect vector and reservoir plant of ‘*Fragaria × ananassa*’ phyllody phytoplasma (16SrXIII-F) in central region of Chile. Plant Dis..

[148] Montano H.G., Davis R.E., Dally E.L., Hogenhout S., Pimentel J.P., Brioso P.S.T. (2001). ‘*Candidatus* Phytoplasma brasiliense’, a new phytoplasma taxon associated with hibiscus witches’ broom disease. Int. J. Syst. Evol. Microbiol..

[149] Ekstein B., Barbosa J.C., Rezende J.A.M., Bedendo I.P. (2011). A *Sida* sp. is a new host for ‘*Candidatus* Phytoplasma brasiliense’ in Brazil. Plant Dis..

[150] Canale M.C., Bedendo I.P. (2013). ‘*Candidatus* Phytoplasma brasiliense’ (16SrXV-A subgroup) associated with cauliflower displaying stunt symptoms in Brazil. Plant Dis..

[151] Wei W., Pérez-López E., Bermúdez-Díaz L., Davis R.E., Granda-Wong C., Zhao Y. (2017). First report of a new grapevine yellows disease in Peru and its association with infection by a ‘*Candidatus* Phytoplasma brasiliense’-related phytoplasma strain. Plant Dis..

[152] Wei W., Pérez-López E., Davis R.E., Bermúdez-Díaz L., Granda-Wong C., Wang J., Zhao Y. (2017). ‘*Candidatus* Phytoplasma brasiliense’-related strains associated with papaya bunchy top disease in Northern Peru represent a distinct geographic lineage. Crops Prot..

